# Importins involved in the nuclear transportation of steroid hormone receptors: *In silico* and *in vitro* data

**DOI:** 10.3389/fendo.2022.954629

**Published:** 2022-09-06

**Authors:** Konstantina Kalyvianaki, Athanasios A. Panagiotopoulos, Maria Patentalaki, Elias Castanas, Marilena Kampa

**Affiliations:** Laboratory of Experimental Endocrinology, School of Medicine, University of Crete, Heraklion, Greece

**Keywords:** nuclear translocation, importins, NLS, steroid receptors, hinge region

## Abstract

The nuclear receptor superfamily (NRS) consists of 48 receptors for lipophilic substances and is divided into 7 different subfamilies, with subfamily 3 comprising steroid hormone receptors. Several nuclear receptors usually bind their cognate ligands in the cytosol and the complex (mono- or dimerized) is transported to the nucleus, where it acts as a transcription initiating factor for a number of genes. The general structure of nuclear receptors consists of an N-terminal activating domain (A/B), important for the binding of activating or inhibitory co-factors, the DNA-binding domain (C), responsible for the association of the receptor-ligand-co-factor complex to the nucleus, the ligand-AF2 domain (E/F), where ligand binding occurs as well as that of ligand-dependent activating/inhibiting factors, and a flexible/non-structured domain (D), linking the DBD and LBD, called hinge region, on which a significant number of post-translational modifications occur. This hinge domain, for the sub-class of steroid receptors, is a non-structured domain and was reported as mainly responsible for the nuclear transport of steroid receptors, since it contains a specific amino acid sequence (Nuclear Localization Signal-NLS), recognized by importin α. In addition to the importin α/β complex, a number of other importins have been discovered and reported to be responsible for the nuclear transport of a number of significant proteins; however, the corresponding recognition sequences for these importins have not been identified. Recently, we have reported the identification of the NLS sequences for importins 4, 5 and 7. In this work, we provide *in silico* data, followed by experimental *in vitro* validation, showing that these alternative importins are responsible for the nuclear transportation of steroid hormone receptors such as ERα, AR and PR, and therefore they may consist of alternative targets for the pharmacological manipulation of steroid hormone actions. Moreover, we provide additional *in* silico data for the hinge region of steroid hormone receptors which is highly enriched with NLS sequences for importins 4, 5 and 7, in addition to the recognition NLS for importin α/β.

## 1 Introduction

With the seminal discovery of Jensen (1) that estradiol might bind to an intracellular protein, the field of nuclear receptors initiated. This discovery was followed by the identification and preliminary characterization of the receptor protein (2, 3) and gene (4). At the same time, the discovery of other hormone receptors genes [reviewed in (5) and references herein] established the notion that all these receptors have a similar structure; this was the onset of the nuclear receptor superfamily group of receptors. With more than 300 receptors, spanning in all animals from porifera to mammals (6), nuclear receptors are involved in a great spectrum of cellular functions, including reproduction and development, lipid metabolism, xenobiotic and bile acid metabolism, and CNS and basal metabolism [(7), http://nrresource.org/education/general-information/nr-functional-classification/], acting as transcription factors (see (5, 8), for reviews and references herein). In humans, 48 different nuclear receptors have been identified (9), classified in seven distinct subfamilies (NR0-6, NR Nomenclature - Nuclear Receptor Resource (nrresource.org)), with Subfamily 3 encoding steroid hormone receptors (NR3A: Estrogen Receptors, NR3B: Estrogen Related (orphan) Receptors, NR3C: 3-ketosteroid (AR, GR, MR) receptors). It is important to note, however, that from the very early time of steroid research, alternative (rapid) effects of steroids have also been identified [(10), also mentioned in the first publication about a putative steroid receptor (1)], a field which took more than 30 years to be acknowledged, but which led to recent interesting findings and physiological implications (the interested reader should consult the proceedings of the Rapid Response to Steroid Hormones – RRSH – biannual meetings, all published in the Journal *Steroids*).

**Figure 1:**
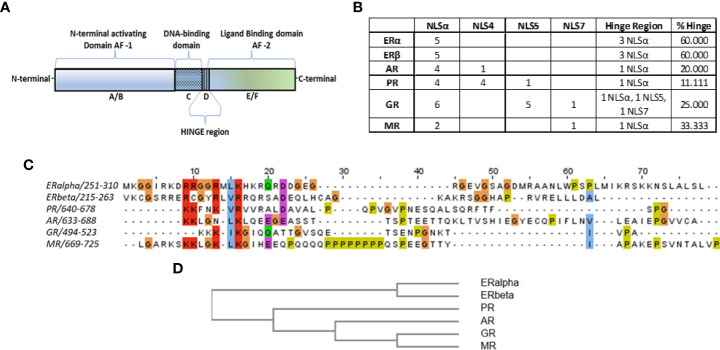
**(A)** Schematic representation of nuclear receptors functional domains. **(B)** Table summarizes the identified importins α, 4, 5 and 7 NLS sequences in the structure of steroid hormone receptors (columns 2-5), according to data presented in **Supplemental Figure 1**. In column 6, the detailed number of putative NLS sequences in the hinge region of each receptor is presented, while in column 7 the percentage of the hinge region-identified NLS over the total NLS sequences is presented. **(C)**Alignment of the hinge regions of steroid hormone receptors, with the MafftWS algorithm (11) in JalView V2.11.2.2 (12). Coloring of amino acids is presented according to the CrustalW color scheme (13) (see http://www.jalview.org/help/html/colourSchemes/clustal.html for details). **(D)** Similarity tree of the hinge region of steroid hormone receptors with the Blosum62 algorithm, performed in JalView.

All nuclear receptors share a common structure, comprising an N-terminal activating domain (A/B), contains the activation function 1 (AF-1) responsible for the binding of activating or inhibitory co-factors, the DNA-binding domain-DBD (C) responsible for the association of the receptor-ligand-co-factor complex with the DNA, the ligand binding domain-LBD (E), where the ligand binding occurs, the activation function 2 (AF-2) domain (F) where ligand-dependent activator/inhibitory factors bind, and a flexible/non-structured domain (D), linking the DBD and LBD, called the hinge region [(5, 6), **Figure 1A**]. As its name implies, the hinge region was considered a linking region of the receptor(s). However, it was later revealed to have several important functions. It is mainly responsible for the nuclear translocation of steroid receptors, since it contains (at the interface between DBD-hinge region) a specific amino acid sequence (Nuclear Localization Signal-NLS), recognized by importin α (14–24). Moreover, as it was found for estrogen receptor alpha (ERα), it possesses an intrinsic activity for calmodulin binding (25) and is the site of a number of post-translational modifications. In addition, we have reported that a fragment of this region [potentially regulated by the intense trafficking of the receptors (26–28)] possesses specific activities, including pro-estrogenic (25), pro-apoptotic (29) actions, while it modulates the migratory activity of human breast cancer cells *in vitro* (30), interacting with specific isoforms of ERα (31).

All receptors share a higher homology in their DBD, as expected, followed by a relatively high homology at their LBD, as all bind lipophilic molecules (32, 33).

Early observations on the mechanistic steps of ER activation revealed the nuclear translocation and dimerization of the receptor (2), after hormone binding (34–37). In parallel, the role of (nuclear) ERs in breast cancer and other endocrine-related tumors had been advanced (38, 39), reviewed in (40). Subsequently, the mode of action and the role of nuclear translocation of nuclear receptors in different hormone actions has been described and pharmacologically exploited (41, 42). However, the proteins and the signals involved in their nuclear translocation have not been fully elucidated.

The cytoplasmic-nuclear transport of proteins is orchestrated by a class of cellular proteins, collectively known as karyopherins, involving exportins, importins and adaptor proteins [see (43) for a review]. Cargo proteins have specific motifs named Nuclear Localization Signals (NLS), responsible and necessary for the identification and the binding of importins. Until recently, only a few NLS motifs were recognized [see references (44, 45) for reviews] for importin α (46) and the M9 NLS (recognized by importin β2, also known as transportin) (47–49). An increasing number of proteins are identified to contain the monopartite classical importin α NLS sequences, KRRR and KRKXK (50–57). Recently, using a bio-informatics approach, based on bibliographic and simulation data, and experimental *in vitro* validation, we have presented the sequence EKRKI(E/R)(K/L/R/S/T) as a recognition motif for binding with Importin 7 (58), a result recently confirmed by another group (59) while recent data [(60) under review] identified the sequences LPPRS(G/P)P and KP(K/Y)LV as recognition motifs for importins 4 and 5 binding, respectively.

In this work, using these new data, we have interrogated the sequences of steroid hormone receptors (NR3 group) for importins’ recognition sequences. We report an abundance of NLS sequences in all receptor structures for importins α, 4, 5 and 7, in the whole span of the receptor molecules. Interestingly, the hinge region of all receptors has 1 to 3 sequences and in combination with its small size and its non-structured conformation renders it as an important element for steroid receptor nuclear translocation. Among the receptors studied, it is to note that ERα and ERβ have only importin α NLSs, while other receptors like AR and PR have NLS sequences for multiple importins.

## 2 Material and methods

### 2.1. Identification of NLS sequences in steroid hormone receptor structures

Amino-acid sequences for ERα and β, PR, AR, GR and MR were retrieved from NCBI (https://www.ncbi.nlm.nih.gov/protein) (#P03372, Q92731, P06401, P10275, P04150 and P08235 respectively). The hinge region in each sequence was retrieved from the same database (see **Supplemental Table 1**). Multiple alignments for each NLS sequence [monopartite Importin α NLS KRRR and KRKXK (50–57), (L)PPRS(G/P)P, KP(K/Y)LV for Importins 4 and 5 respectively (60) and EKRKI(E/R)(K/L/R/S/T) for importin 7 (58)] were identified with the online Clustal Omega tool on the EMBL-EBI server (https://www.ebi.ac.uk/Tools/msa/clustalo/) (61, 62). Only sequences with at least 50% homology in the amino acids of each NLS were retained. Hinge regions alignment was constructed, analyzed and visualized in JalView v2.11.1.7 (12).

### 2.2. Bio-informatics detection of active and inactive NLS sequences

Due to the presence of a large number of unstructured regions in the sequence of steroid hormone receptors, their crystallization has not been described. However, fragments (especially of their E-F regions (binding domains and AF2) have been reported. Recently, using AI methods, a prediction of ~1000000 protein 3D structure was reported (AlphaFold and AlphaFold2 (63–65). Here, we have used the crystal structures of the C-terminal domain of the steroid hormone receptors, bound to an agonist or antagonist, and the structural prediction of domains A-C (AF-1 and DBD) from the AlphaFold protein structure database (https://alphafold.ebi.ac.uk/). The same database was used for the structure of the unliganded LBD-AF-2 domain (see **Supplemental Table 2**). For each identified steroid hormone receptor fragment, we have manually annotated the NLS sequence we have identified (see previous paragraph) and reported it as “active” if it is located at the surface of the protein (and therefore accessible to importins) or as “inactive” if not (see Results and **Supplemental Figures 3–8**, for further details). In the hinge region, all NLS were characterized as “active”, as this part of the receptor molecule is unstructured and therefore accessible for an interaction with importins.

The validity of the AlphaFold2 solutions were verified by comparing them to data obtained with the Galaxy Web server (66–69) (https://galaxy.seoklab.org/cgi-bin/submit.cgi?type=TBM), while the ERRAT2 score (70) (https://saves.mbi.ucla.edu/) was <80%, suggesting a valid prediction (**Supplemental Figure 2**).

### 2.3. Experimental validation of bio-informatic data

#### 2.3.1 Cell lines and culture conditions

T47D and LNCaP cell lines were purchased from DSMZ (Braunschweig, Germany), and were cultured in RPMI-1640 (Gibco™, Thermo Fisher Scientific) supplemented with 10% Fetal Bovine Serum (Qualified, Gibco™, Thermo Fischer Scientific), at 37°C and 5% CO_2_. The selection of the specific cell lines was determined due to their levels of expression of Progesterone Receptor (PR), Estrogen Receptor alpha (ERα) for T47D cells and Androgen Receptor (AR) for LNCaP and T47D cells (CCLE, Cancer Cell Line Encyclopedia database (https://sites.broadinstitute.org/ccle/ ) (71). Expression levels of importin α, 4, and 5 for the specific cell lines, were also taken into consideration (The Human Protein Atlas, https://www.proteinatlas.org/ (72) and our results). All media were purchased from Fisher Scientific and all chemicals from Sigma (St. Louis, MO), unless otherwise stated.

#### 2.3.2 Transfection method for importins (IPOs) silencing

Cells were seeded at an initial of 5 × 10^5^ cells/well in a 6- well plate with 1 ml medium per well and incubated for 24 hours. Attractene Transfection Reagent (QIAGEN) was used, according to standard protocols, to transfect the cells with the specific siRNAs (0.05 μg siRNA and 0.2 μl Attractene Transfection Reagent/10^4^ cells) for IPO4 (AM16708, ID: 109561), IPO5 (AM16708, ID: 106742), IPOA1 (AM16708, ID: 11126) or scrambled siRNAs (AM16708, ID: 149158) (Thermo Fischer Scientific, Waltham, MA USA). After 24 h, fresh medium was added and 24 h later cells were collected and analyzed or fixed with 4% paraformaldehyde in PBS for 10 min.

#### 2.3.3 RNA isolation and real time PCR

Transfection efficiency was evaluated by measuring the receptor gene expression through real-time quantitative PCR (real-time qPCR). Total cell RNA from the 6 well plates was isolated using the RNA isolation Kit (Nucleospin, Macherey-Nagel, DE), cDNA was synthesized using the PrimeScript™ RT Kit (TaKaRa Bio Inc) and real time PCR was performed using the KAPA SYBR FAST qPCR Master Mix (Kapa Biosystems, Inc. Wilmington, MA, USA) as previously described (73). The following primer pairs (synthesized by Eurofins Genomics, Ebersberg, Germany) were used (5’->3’): IPO4, forward ACGGAACAGCTCCAGATCGT, reverse AGCAAAAGCCCCATCTCTCTC, IPO5, forward CTGCTGAAGAGGCTAGACAAATG, reverse TCTGCCGCAATATCACAAACTT, IPOA1, forward ATTGCAGGTGATGGCTCAGT, reverse CTGCTCAACAGCATCTATCG and Cyclophilin A, forward ATGGTCAACCCCACCGTGT, reverse TTCTGCTGTCTTTGGAACTTTGTC. In all cases transfection efficiency ranged between 40% to 70% (**Supplemental Figure 10**).

#### 2.3.4 Immunofluorescence-confocal microscopy and analysis of data

Paraformaldehyde fixed cells were incubated with blocking buffer containing Triton X-100 0.2% w/v for 10 min and were stained using primary antibodies against ERα, AR, PR and nuclear envelope lamins (for labelling of the nucleus) and fluorescently labelled secondary antibodies (see **Supplemental Table 3** for the specific antibodies and dilutions used). Initially cells (pretreated for 90 min with 10^-7^ M DHT for AR or ORG 2058 for PR and untreated for ERα) were washed with PBS and incubated with the primary antibodies for AR, PR or ERα respectively for 1hr at RT, followed by 2 PBS washes and a 45min staining with the fluorescent secondary antibodies. Afterwards cells were incubated for 45 min with a primary antibody for lamin B1 or A/C (nuclear envelope markers) at RT, washed with PBS (x2) and stained for 45 min with the appropriate secondary antibody. Fixed-stained cells were mounted with Mounting Medium (Inova Diagnostics, Inc, San Diego) containing DAPI and observed on an inverted confocal scanning microscope (Leica SP5) using a 63× objective lens with oil immersion and an optical zoom 2x.

The fluorescence intensity ratio of each receptor (ERα, PR and AR) in the nucleus and the cytoplasm was quantified using the Image J software (https://imagej.nih.gov/). The area (nucleus or cytoplasm) in the cell of interest was selected using the polygon selection tool and measurements of different variables were taken. To calculate the corrected total cell fluorescence (CTCF) the following formula was used: CTCF = Integrated Density – (Area of selected cell X Mean fluorescence of background readings). For the mean background readings ten measurements from ten different regions next to the cells were taken. The ratio of the fluorescence intensity of the cytoplasmic region to the nucleus quantifies the nuclear translocation of each receptor. At least 50 cells per condition were analyzed from at least 3 independent experiments. GraphPad Prism 8.0.1 (GraphPad Software Inc. San Diego CA) was used for parametric statistical analysis and results were displayed as mean ± SEM. p-values < 0.05 were considered statistically significant.

## 3 Results

### 3.1. Identification of importins α, 4, 5 and 7 recognition signals in the sequence of steroid hormone receptors by a bio-informatic approach

Blast sequence alignment of the NLS sequences for importins α, 4, 5 and 7 on steroid hormone receptors (ERα and β, PR, AR, GR, MR) (**Supplemental Figure 1**), identified a receptor-specific pattern of NLS recognition sequences summarized in **Figure 1B**. Interestingly, for ERα and β, the only identified NLS sequences were those for importin alpha, suggesting that this is the only importin responsible for the nuclear import of the receptor. For the androgen receptor (AR) the main NLS sequences (4/5) recognize importin α, while an additional sequence (located at the boundary of AF-1 and DBD (see **Supplemental Figure 1**) recognizes importin 4. For the PR, we have identified abundant NLS sequences (4 for importin α, 4 for importin 4 and 1 for importin 5). In the glucocorticoid receptor (GR) we have identified 12 NLS sequences, 6 for importin α, 5 for importin 5 and 1 for importin 7. Finally, at the structure of the mineralocorticoid receptor, we have identified 3 NLS sequences (2 for importin α and 1 for importin 7). These data provide novel evidence for the possible role of different importins (α, 4, 5 and 7), in the ligand-dependent and -independent nuclear translocation of liganded and unliganded steroid hormone receptors. In addition, our data confirm the primary role of importin α, for the nuclear translocation of all steroid hormone receptors.

We have further concentrated on the analysis of the hinge region of steroid hormone receptors. This short, unstructured sequence of 40-60 amino acids, analyzed extensively especially for ERα, has been reported to possess an intrinsic activity by binding calmodulin (25) and being the site of a number of post-translational modifications. Alignment and clustering of this region (**Figures 1C, D**) revealed a very good match for ERα and β on one hand, and for GR and MR on the other. Furthermore, 60% of ERα and β NLSs for importin α, were located in the hinge region, positioning it as a primary part of the receptor involved in its nuclear translocation. This percentage was lower in other steroid receptors (11% in PR, 20% in AR, 25% in GR and finally 33% in MR of total NLS-identified sites MR). It is important to note that importin selectivity also varies among the different steroid hormone receptors, although all possess recognition motifs for importin α and only GR hinge region expresses sequences for importins 5 and 7 (**Figure 1B**). In this respect, we provide an additional hint for the significance of the hinge region, heavily involved in the nuclear transport of the steroid hormone receptors.

### 3.2. Validation of the identified NLS-Importins’ role in steroid receptors’ nuclear translocation

#### 3.2.1. *In silico*

The recognition signals and the importins that were identified in the different steroid receptors were initially validated *in silico* by examining their position in each receptor 3D structure in order to find their accessibility for binding to importins. If they are present at the surface of each receptor 3D structure, they are characterized as “active” (i.e. accessible to importins for binding). In contrast, when they are buried in the structure, away from the surface, they are inaccessible to importins and characterized as “inactive”. Both liganded, unliganded and non-liganded LBD receptor structures were examined. The great majority of the NLSs was found either in non-structured regions of the receptor molecules, or at the surface of structured regions, accessible to importins for binding and therefore characterized as active (**Supplemental Figures 3–8**). We have used the AlphaFold2 PDB files as templates (64, 65), for the characterization of NLS sequences in the A-C N-terminal part of the receptors. As expected, all sites, situated in the non-structured regions of the receptors are active, while a receptor-dependent active or inactive sites are identified in the structured parts of the N-terminal regions (see **Figure 1B**, **Supplemental Figures 1**, **3–8**). Structured data of the AlphaFold solution (https://alphafold.ebi.ac.uk/), was verified through an *ab initio* 3D conformation in the Galaxy Server (https://galaxy.seoklab.org/cgi-bin/submit.cgi?type=TBM) (66–69) showing a very good match (RMSD<2Å in all cases), and the ERRAT program (**Supplemental Figure 2**) (70). Data concerning the hinge region (a preferential site for all NLS sequences), which is non-structured and therefore all identified sites are labeled as active, are also presented in **Figures 1B–D** (please see the previous paragraph and Discussion section for additional details). Interestingly, no NLS was identified in ERα, ERβ and MR LBD, suggesting that their nuclear translocation (at least through importin α, is independent of ligand binding **Supplemental Figures 3, 4, 8**). Androgen agonists and antagonists binding to the AR do not modify the active NLS identified at the LBD (**Supplemental Figure 5**). In contrast, one of the two identified NLS (for importin α binding) in the LBD of the PR is inactive after agonist binding and active in the unliganded and antagonist-bound form (**Supplemental Figure 6**), while for the GR the one NLS of the LBD is predicted as always accessible to importin 5 (**Supplemental Figure 7**).

#### 3.2.2. *In vitro*

**Figure 2:**
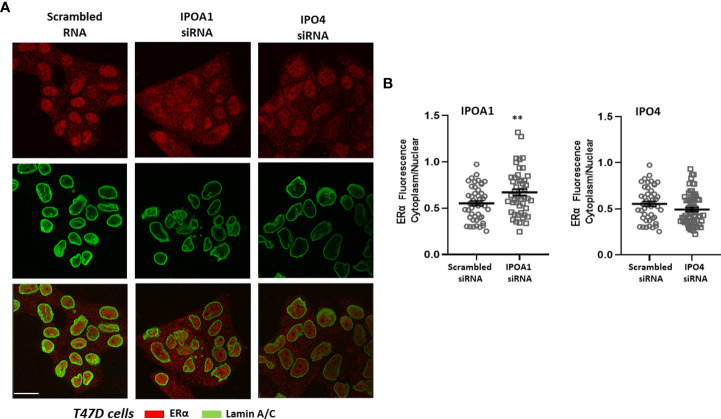
**(A)** Representative confocal pictures of T47D cells stained for estrogen receptor alpha (ERα) (red) and lamin A/C (green) (3^rd^ raw is their overlay). T47D cells were either transfected with a scrambled siRNA or a specific siRNA for importin α (IPOA1) or importin 4 (IPO4), used as a negative control, as our bioinformatics approach did not identify any importin 4 NLS. Magnification x1260 (scale bar, 40 μm). **(B)** Intensity of fluorescence in the cytoplasm and nucleus was quantified (see Material and Methods for details) in at least 50 cells per condition from 4 independent experiments (n=4) and is given as the Cytoplasm/Nuclear fluorescence ratio comparing cell with specific IPOA1 siRNA to those with the scrambled siRNA. ** denotes statistical significance P< 0.01.

**Figure 3:**
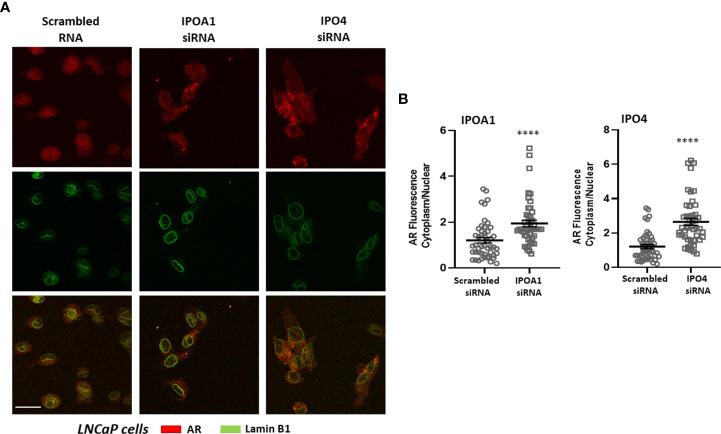
**(A)** Representative confocal pictures of LNCaP cells stained for androgen receptor (AR) (red) and lamin B1 (green) (3^rd^ raw is their overlay). LNCaP cells were transfected either with a scrambled siRNA or a specific siRNA for importin α (IPOA1) or importin 4 (IPO4) and treated with DHT (10^-7^M) for 90 min to ensure nuclear localization. Magnification x1260 (scale bar, 40 μm). **(B)** Intensity of fluorescence in the cytoplasm and nucleus was quantified (see Material and Methods for details) in at least 50 cells from 4 independent experiments (n=4) and is given as the Cytoplasm/Nuclear fluorescence ratio in each condition. **** denotes statistical significance P< 0.0001.

**Figure 4:**
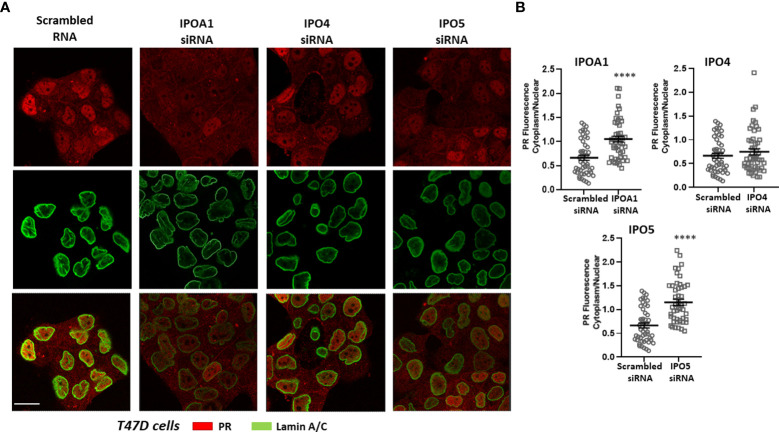
**(A)** Representative confocal pictures of T47D cells stained for progesterone receptor (PR) (red) and lamin A/C (green) (3^rd^ raw is their overlay). T47D cells were either transfected with a scrambled siRNA or a specific siRNA for importin α (IPOA1), importin 4 (IPO4) or Importin 5 (IPO5) treated with ORG-2058 (10^-7^M) for 90 min to ensure nuclear localization. Magnification x1260 (scale bar, 40 μm). **(B)** Intensity of fluorescence in the cytoplasm and nucleus was quantified (see Material and Methods for details) in at least 50 cells from 3 independent experiments (n=3) and is given as the Cytoplasm/Nuclear fluorescence ratio in each condition. **** denotes statistical significance P< 0.0001.

The role of the *in silico* identified importins for the nuclear transportation of the steroid receptors was also validated *in vitro*. The nuclear presence of three different receptors, ERα, AR and PR, was examined in cells expressing the specific importins (**Supplemental Table 4**) in the absence or presence of specific siRNAs that inhibit their expression (see **Supplemental Figure 10** for the effect of each siRNA in each cell line). As it is shown in **Figure 2**, nuclear ERα is significantly decreased in T47D cells transfected with IPOA1 siRNA (for importin α), as expressed by an increase in the ratio of cytoplasmic/nuclear receptor localization. Such an effect was not observed when IPO4 siRNA (for importin 4) was utilized (**Figure 2**), verifying the *in silico* data that only importin α NLSs are present in the ERα sequence (**Figure 1B** and **Supplemental Figure 1**). For AR both importins α and 4 NLSs were identified *in silico*; in LnCaP (**Figure 3**) and T47D cells (**Supplemental Figure 9**) we show that, reducing the expression of either importin with specific siRNAs, significantly decreased the nuclear localization of AR. Finally, for the PR, we have found NLS for importin α, 4 and 5 (**Figure 1B** and **Supplemental Figure 1**); in T47D cells, the IPOA1 and IPO5 siRNAs were able to significantly decrease nuclear PR translocation (**Figure 4**). Surprisingly, IPO4 knocking down, with a specific siRNA, did not significantly inhibit PR transfer to the nucleus (**Figure 4B**). It seems therefore that importin 4 might have not a significant role in PR nuclear transportation, in T47D cells (in contrast to AR), a result that needs further investigation.

## 4 Discussion

It is now widely accepted that steroid hormone receptors in order to fulfill their role as transcription factors, must translocate (either in their free or ligand-bound form) from the cytoplasm to the nucleus. Importins seem to play a significant role and based on this a sustained research effort for their interactions was initiated.

For ERα, nuclear accumulation is mediated through an interaction with importin α (14, 17, 23) through a specific NLS, at the interface of DBD and hinge region (amino acids 266-269, a result in accordance with our findings) and importin 3 (23), or passive transport (23, 74). It was also noted that untreated (unliganded ERα) or SERM-treated breast cancer cells (tamoxifen-ERα) show a diffuse nuclear staining, while agonist-treated cells (estradiol-ERα) form numerous nuclear focal accumulations (75). For ERβ, although there is a great homology at the DBD-hinge region, where the NLS for importin α in ERα exists, no specific mechanisms have been identified but only a diffusion through nucleoporin 153, in association with eNOS (76) has been reported.

For the androgen receptor (AR), nuclear translocation relies also on importin α (20, 21), while the nuclear localization signal that the authors reported resides at the junction of the DBD and the hinge region (amino acids 617-635). This signal is also conserved in the structure of the progesterone, mineralocorticoid and glucocorticoid receptors (amino acids 625-643, 661-679 and 479-497 respectively, an element also reported here) (20). In addition, the glucocorticoid receptor (GR) translocates through additional interaction with importin 7 (58, 77), a NLS comprised in the LBD of the receptor (78) and identified here as a recognition motif for importin 5, and importin 13 (79).

Progesterone receptor (PR) was reported to interact with importin α at the DBD-hinge interface (15, 16, 18, 20) and a ligand-dependent NLS at the DBD (16) that was also identified here as an NLS for importin α. In addition, another NLS at the LBD (15) has been described, which recognizes importin 4 (amino acids 685-689) or importin 5 (amino acids 781-784 and 927-930), as reported here. Finally, the mineralocorticoid receptor (MR) interacts with importin α (19, 20, 22, 24). Moreover, two additional NLS sequences have been identified, one in the DBD and another in the LBD of the receptor (24), which, as reported here, are recognition sequences for importin 7.

Even though nuclear translocation is a necessity for steroid hormone receptors’ action, as mentioned above, a limited number of NLS sequences were previously identified. Here we extend this knowledge, reporting a higher number of putative NLS sequences, expanding to the whole length of the receptors, which are active in both the free and their ligand-bound forms. In this respect, the recognition of the receptor by karyopherins is redundant, presenting a repetition of recognition motifs for the same importin (with importin α having the largest number of motifs in all receptors examined), and complementary, expressing NLS sequences for multiple importins, in all receptors, with the exception of ERα, ERβ and the MR. Moreover, our *in vitro* data have verified the importance of the different, *in silico* identified, NLS- importins, for the nuclear translocation of three different steroid receptors (ERα, AR and PR) and revealed the significance of importin α and the specific roles of importins 4 and 5, for the receptors’ nuclear translocation. We have to mention, however, that importin 4 seems not to play a significant role for PR nuclear translocation in T47D cells, in contrast to its role for the nuclear transfer of AR, in both T47D and LnCaP cells, an element which needs further investigation. Therefore, our results (both *in silico* and *in vitro*) confirm the primary role of importin α, as the primary karyopherin involved in the cytoplasmic-nuclear shuttle of steroid hormone receptors, in accordance with previous data (14–24). However, further research including mutagenesis experiments, is required for confirming our findings and to better determine the importance and function of each identified NLS motif, as the binding of co-regulatory molecules, or HSP might mask some of these sites. Nevertheless, our *in silico* data, presented here suggest that LBD sites are, in their great majority, active in both the unliganded and liganded AR, PR and GR receptors. In addition, as derived from our data, even in the event of mutations in one part of the receptor, it is rather improbable that the molecule will be sequestrated in the cytoplasm.

For all steroid receptor molecules, the interface between DBD-hinge region has a sequence (NLS) recognized by importin α (14–24), a result also reported here. This finding pinpoints the hinge region as a primary interface for importin α recognition, and stress its role for steroid hormone receptor nuclear translocation. In addition, the non-structured conformation of this region ensures that the NLS sequence is always accessible for importin binding. The hinge region has emerged from an accessory part of the receptor, linking the DBD to the LBD parts, to a site of great importance, involved in post-translational modifications and co-regulators (such as calmodulin) binding [see (25), for a discussion]. We have therefore analyzed this region for all steroid hormone receptors. Alignment of all hormone receptor hinge sequences identified two parts: the N-terminal part, which presents the greater homology and expresses the conserved importin α NLS, and a C-terminal part, which is receptor-specific, and shows a limited homology among the different receptors. Interestingly, clustering of the hinge regions revealed a greater homology between the two ERs (ERα and ERβ), and the three 3-ketosteroid receptors (AR, GR and MR), with PR the other 3-ketosteroid receptor, being positioned between the two groups. This result parallels the evolution of steroid receptors in vertebrates, reported by Thornton (80), from an initial functional ER, followed by the apparition of PR and the other 3-ketosteroid receptors in that order. In addition, we have identified in this hinge region a number of additional putative NLS sequences for different importins, in a receptor-specific manner, ranging from 11 to 60% of all identified sites. In this respect, the hinge region expresses a significantly higher number of NLS sequences (from ~3 to 6 times, as compared to the length of this region as compared to the total length of each receptor molecule) and verifies its importance for the nuclear translocation of the receptors.

Interestingly our group has identified, at the C-terminal part of the ERα hinge region (amino acids 295-311) a decaheptapeptide, named ERalpha17p, which can be released after proteasomal degradation of ERα and possesses estrogenic (25, 27) and pro-apoptotic (29) actions and modulates the migratory activity of human breast cancer cells *in vitro* (30) by interacting with specific isoforms of ERα (31). It is to note, that this peptide induces the dissociation of HSP70 from ER, having a trophic effect on ERα^+^ breast cancer cells (81), and exhibits also a proper transcriptional activity, acting in an estrogen receptor-isoform-related and unrelated manner (31). Although, due to its size, a passive diffusion through the nuclear pores is possible (74), the identification of an importin α NLS in its sequence suggests an additional regulated nuclear translocation, possibly important for its proper transcriptional activity (31) or other nuclear actions.

In conclusion, the findings of the present work clearly show that steroid receptors in order to fulfill their role as transcription factors have a large number of NLS sequences ensuring their translocation to the nucleus. These sequences expand to the whole length of the receptors with the hinge region being highly enriched. Importin α has the largest number of motifs in all receptors examined. However apart from importin α, other importins seem to play a significant role in their transport to the nucleus. Therefore, if our findings are further confirmed they may represent alternative targets for the pharmacological manipulation of diseases relating to steroid hormone action.

## Data availability statement

The original contributions presented in the study are included in the article/**Supplementary Material**. Further inquiries can be directed to the corresponding authors.

## Author contributions

Conceived and designed the study: MK and EC. Performed the analyses and experiments: KK, AP, and MP. Wrote the paper: MK and EC. All authors read and approved the final manuscript.

## Funding

This work was partially supported by Greece and the European Union (European Social Fund- ESF) through the Operational Programme (Human Resources Development, Education and Lifelong Learning) in the context of the project "Strengthening Human Resources Research Potential via Doctorate Research" (MIS-5000432), implemented by the State Scholarships Foundation (IKY) to AP (PhD scholarship), a Special Fund for Research Grants (ELKE) of the University of Crete to MK and KK and by the Hellenic Foundation for Research and Innovation (H.F.R.I.) under the “First Call for H.F.R.I. Research Projects to support Faculty members and Researchers and the procurement of high-cost research equipment grant” (Project Number: 3725 to MK).

## Conflict of interest

The authors declare that the research was conducted in the absence of any commercial or financial relationships that could be construed as a potential conflict of interest.

## Publisher’s note

All claims expressed in this article are solely those of the authors and do not necessarily represent those of their affiliated organizations, or those of the publisher, the editors and the reviewers. Any product that may be evaluated in this article, or claim that may be made by its manufacturer, is not guaranteed or endorsed by the publisher.

## Abbreviations

ER, Estrogen Receptor; PR, Progesterone Receptor; AR, Androgen Receptor; GR, Glucocorticoid Receptor; MR, Mineralocorticoid Receptor; NLS, Nuclear Localization Signal; IPO, Importin.

## References

[1] Jensen EV. On the mechanism of estrogen action. Perspect Biol Med (1962) 6:47–59. doi: 10.1353/pbm.1963.0005 13957617

[2] Toft D Gorski J. A receptor molecule for estrogens: Isolation from the rat uterus and preliminary characterization. Proc Natl Acad Sci USA (1966) 55:1574–81. doi: 10.1073/pnas.55.6.1574 PMC224361 5227676

[3] Toft D Shyamala G Gorski J. A receptor molecule for estrogens: Studies using a cell-free system. Proc Natl Acad Sci USA (1967) 57:1740–3. doi: 10.1073/pnas.57.6.1740 PMC224541 5232110

[4] Green S Walter P Kumar V Krust A Bornert JM Argos P. Human oestrogen receptor cDNA: Sequence, expression and homology to v-erb-A. Nature (1986) 320:134–9. doi: 10.1038/320134a0 3754034

[5] Mazaira GI Zgajnar NR Lotufo CM Daneri-Becerra C Sivils JC Soto OB. The nuclear receptor field: A historical overview and future challenges. Nucl Receptor Res (2018) 5:1–21. doi: 10.11131/2018/101320 PMC6108593 30148160

[6] Sladek FM. What are nuclear receptor ligands? Mol Cell Endocrinol (2011) 334:3–13. doi: 10.1016/j.mce.2010.06.018 20615454 PMC3010294

[7] Vroling B Thorne D Mcdermott P Joosten HJ Attwood TK Pettifer S. NucleaRDB: information system for nuclear receptors. Nucleic Acids Res (2012) 40:D377–380. doi: 10.1093/nar/gkr960 PMC3245090 22064856

[8] Frigo DE Bondesson M Williams C. Nuclear receptors: from molecular mechanisms to therapeutics. Essays Biochem (2021) 65:847–56. doi: 10.1042/EBC20210020 PMC8628184 34825698

[9] Alexander SP Cidlowski JA Kelly E Mathie A Peters JA Veale EL. THE CONCISE GUIDE TO PHARMACOLOGY 2021/22: Nuclear hormone receptors. Br J Pharmacol (2021) 178 Suppl 1:S246–63. doi: 10.1111/bph.15540 PMC9513947 34529827

[10] Szego CM Davis JS. Adenosine 3',5'-monophosphate in rat uterus: acute elevation by estrogen. Proc Natl Acad Sci USA (1967) 58:1711–8. doi: 10.1073/pnas.58.4.1711 PMC223984 4295833

[11] Katoh K Toh H. Parallelization of the MAFFT multiple sequence alignment program. Bioinformatics (2010) 26:1899–900. doi: 10.1093/bioinformatics/btq224 PMC2905546 20427515

[12] Waterhouse AM Procter JB Martin DM Clamp M Barton GJ. Jalview version 2–a multiple sequence alignment editor and analysis workbench. Bioinformatics (2009) 25:1189–91. doi: 10.1093/bioinformatics/btp033 PMC2672624 19151095

[13] Larkin MA Blackshields G Brown NP Chenna R Mcgettigan PA Mcwilliam H. Clustal W and clustal X version 2.0. Bioinformatics (2007) 23:2947–8. doi: 10.1093/bioinformatics/btm404 17846036

[14] Picard D Kumar V Chambon P Yamamoto KR. Signal transduction by steroid hormones: Nuclear localization is differentially regulated in estrogen and glucocorticoid receptors. Cell Regul (1990) 1:291–9. doi: 10.1091/mbc.1.3.291 PMC361473 2100202

[15] Guiochon-Mantel A Lescop P Christin-Maitre S Loosfelt H Perrot-Applanat M Milgrom E. Nucleocytoplasmic shuttling of the progesterone receptor. EMBO J (1991) 10:3851–9. doi: 10.1002/j.1460-2075.1991.tb04954.x PMC453122 1935904

[16] Guiochon-Mantel A Loosfelt H Lescop P Christin-Maitre S Perrot-Applanat M Milgrom E. Mechanisms of nuclear localization of the progesterone receptor. J Steroid Biochem Mol Biol (1992) 41:209–15. doi: 10.1016/0960-0760(92)90346-K 1562504

[17] Ylikomi T Bocquel MT Berry M Gronemeyer H Chambon P. Cooperation of proto-signals for nuclear accumulation of estrogen and progesterone receptors. EMBO J (1992) 11:3681–94. doi: 10.1002/j.1460-2075.1992.tb05453.x PMC556828 1327748

[18] Guiochon-Mantel A Milgrom E. Cytoplasmic-nuclear trafficking of steroid hormone receptors. Trends Endocrinol Metab (1993) 4:322–8. doi: 10.1016/1043-2760(93)90074-O 18407179

[19] Piwien Pilipuk G Vinson GP Sanchez CG Galigniana MD. Evidence for NL1-independent nuclear translocation of the mineralocorticoid receptor. Biochemistry (2007) 46:1389–97. doi: 10.1021/bi0621819 17260968

[20] Cutress ML Whitaker HC Mills IG Stewart M Neal DE. Structural basis for the nuclear import of the human androgen receptor. J Cell Sci (2008) 121:957–68. doi: 10.1242/jcs.022103 18319300

[21] Kaku N Matsuda K Tsujimura A Kawata M. Characterization of nuclear import of the domain-specific androgen receptor in association with the importin alpha/beta and ran-guanosine 5'-triphosphate systems. Endocrinology (2008) 149:3960–9. doi: 10.1210/en.2008-0137 PMC2488236 18420738

[22] Hernandez-Diaz I Giraldez T Arnau MR Smits VA Jaisser F Farman N. The mineralocorticoid receptor is a constitutive nuclear factor in cardiomyocytes due to hyperactive nuclear localization signals. Endocrinology (2010) 151:3888–99. doi: 10.1210/en.2010-0099 20484457

[23] Moriyama T Yoneda Y Oka M Yamada M. Transportin-2 plays a critical role in nucleocytoplasmic shuttling of oestrogen receptor-alpha. Sci Rep (2020) 10:18640. doi: 10.1038/s41598-020-75631-3 33122699 PMC7596556

[24] Grossmann C Almeida-Prieto B Nolze A Alvarez de la Rosa D. Structural and molecular determinants of mineralocorticoid receptor signalling. Br J Pharmacol (2021) 179(13):3103–18. doi: 10.1111/bph.15746 34811739

[25] Gallo D Jacquemotte F Cleeren A Laios I Hadiy S Rowlands MG. Calmodulin-independent, agonistic properties of a peptide containing the calmodulin binding site of estrogen receptor alpha. Mol Cell Endocrinol (2007) 268:37–49. doi: 10.1016/j.mce.2007.01.012 17316976

[26] Leclercq G Lacroix M Laios I Laurent G. Estrogen receptor alpha: impact of ligands on intracellular shuttling and turnover rate in breast cancer cells. Curr Cancer Drug Targets (2006) 6:39–64. doi: 10.2174/156800906775471716 16475975

[27] Gallo D Haddad I Laurent G Vinh J Jacquemotte F Jacquot Y. Regulatory function of the P295-T311 motif of the estrogen receptor alpha - does proteasomal degradation of the receptor induce emergence of peptides implicated in estrogenic responses? Nucl Recept Signal (2008) 6:e007. doi: 10.1621/nrs.06007 18432312 PMC2329824

[28] Leclercq G Gallo D Cossy J Laios I Larsimont D Laurent G. Peptides targeting estrogen receptor alpha-potential applications for breast cancer treatment. Curr Pharm Des (2011) 17:2632–53. doi: 10.2174/138161211797416048 21728983

[29] Pelekanou V Kampa M Gallo D Notas G Troullinaki M Duvillier H. The estrogen receptor alpha-derived peptide ERalpha17p (P(295)-T(311)) exerts pro-apoptotic actions in breast cancer cells *in vitro* and *in vivo*, independently from their ERalpha status. Mol Oncol (2011) 5:36–47. doi: 10.1016/j.molonc.2010.11.001 21163714 PMC5528276

[30] Kampa M Pelekanou V Gallo D Notas G Troullinaki M Pediaditakis I. ERalpha17p, an ERalpha P295 -T311 fragment, modifies the migration of breast cancer cells, through actin cytoskeleton rearrangements. J Cell Biochem (2011) 112:3786–96. doi: 10.1002/jcb.23309 21826705

[31] Notas G Kampa M Pelekanou V Troullinaki M Jacquot Y Leclercq G. Whole transcriptome analysis of the ERalpha synthetic fragment P295-T311 (ERalpha17p) identifies specific ERalpha-isoform (ERalpha, ERalpha36)-dependent and -independent actions in breast cancer cells. Mol Oncol (2013) 7:595–610. doi: 10.1016/j.molonc.2013.02.012 23474223 PMC5528475

[32] Weikum ER Liu X Ortlund EA. The nuclear receptor superfamily: A structural perspective. Protein Sci (2018) 27:1876–92. doi: 10.1002/pro.3496 PMC6201731 30109749

[33] Papageorgiou L Shalzi L Efthimiadou A Bakopoulou F Chrousos G Eliopoulos E. Conserved functional motifs of the nuclear receptor superfamily as potential pharmacological targets. Int J Epigenet (2021) 1:1–10. doi: 10.3892/ije.2021.3

[34] O'malley BW Means AR. Female steroid hormones and target cell nuclei. Science (1974) 183:610–20. doi: 10.1126/science.183.4125.610 4359082

[35] Baulieu EE. Some aspects of the mechanism of action of steroid hormones. Mol Cell Biochem (1975) 7:157–74. doi: 10.1007/BF01731406 168483

[36] Katzenellenbogen BS Ferguson ER. Antiestrogen action in the uterus: Biological ineffectiveness of nuclear bound estradiol after antiestrogen. Endocrinology (1975) 97:1–12. doi: 10.1210/endo-97-1-1 166821

[37] Farookhi R Sonnenschein C. Estrogen-binding parameters of cytoplasmic and nuclear receptors in an established rat endometrial cell line and tumor. Endocr Res Commun (1976) 3:1–19. doi: 10.3109/07435807609057737 179777

[38] Jensen EV Block GE Smith S Kyser K Desombre ER. Estrogen receptors and breast cancer response to adrenalectomy. Natl Cancer Inst Monogr (1971) 34:55–70. 5140877

[39] Leclercq G Heuson JC Deboel MC Mattheiem WH. Oestrogen receptors in breast cancer: a changing concept. Br Med J (1975) 1:185–9. doi: 10.1136/bmj.1.5951.185 PMC1672156 163111

[40] Jensen EV Jordan VC. The estrogen receptor: a model for molecular medicine. Clin Cancer Res (2003) 9:1980–9. doi: 10.1353/pbm.1963.0005 12796359

[41] Burris TP Solt LA Wang Y Crumbley C Banerjee S Griffett K. Nuclear receptors and their selective pharmacologic modulators. Pharmacol Rev (2013) 65:710–78. doi: 10.1124/pr.112.006833 PMC11060414 23457206

[42] Zhao L Zhou S Gustafsson JA. Nuclear receptors: Recent drug discovery for cancer therapies. Endocr Rev (2019) 40:1207–49. doi: 10.1210/er.2018-00222 30869771

[43] Cagatay T Chook YM. Karyopherins in cancer. Curr Opin Cell Biol (2018) 52:30–42. doi: 10.1016/j.ceb.2018.01.006 29414591 PMC5988925

[44] Chook YM Suel KE. Nuclear import by karyopherin-betas: recognition and inhibition. Biochim Biophys Acta (2011) 1813:1593–606. doi: 10.1016/j.bbamcr.2010.10.014 PMC3135726 21029754

[45] Soniat M Chook YM. Nuclear localization signals for four distinct karyopherin-beta nuclear import systems. Biochem J (2015) 468:353–62. doi: 10.1042/BJ20150368 26173234

[46] Kalderon D Roberts BL Richardson WD Smith AE. A short amino acid sequence able to specify nuclear location. Cell (1984) 39:499–509. doi: 10.1016/0092-8674(84)90457-4 6096007

[47] Izaurralde E Jarmolowski A Beisel C Mattaj IW Dreyfuss G Fischer U. A role for the M9 transport signal of hnRNP A1 in mRNA nuclear export. J Cell Biol (1997) 137:27–35. doi: 10.1083/jcb.137.1.27 9105034 PMC2139861

[48] Bogerd HP Benson RE Truant R Herold A Phingbodhipakkiya M Cullen BR. Definition of a consensus transportin-specific nucleocytoplasmic transport signal. J Biol Chem (1999) 274:9771–7. doi: 10.1074/jbc.274.14.9771 10092666

[49] Iijima M Suzuki M Tanabe A Nishimura A Yamada M. Two motifs essential for nuclear import of the hnRNP A1 nucleocytoplasmic shuttling sequence M9 core. FEBS Lett (2006) 580:1365–70. doi: 10.1016/j.febslet.2006.01.058 16455081

[50] Robbins J Dilwortht SM Laskey RA Dingwall C. Two interdependent basic domains in nucleoplasmin nuclear targeting sequence: Identification of a class of bipartite nuclear targeting sequence. Cell (1991) 64:615–23. doi: 10.1016/0092-8674(91)90245-T 1991323

[51] Adam SA. Transport pathways of macromolecules between the nucleus and the cytoplasm. Curr Opin Cell Biol (1999) 11:402–6. doi: 10.1016/S0955-0674(99)80056-8 10395552

[52] Fontes MRM Teh T Kobe B. Structural basis of recognition of monopartite and bipartite nuclear localization sequences by mammalian importin-α11Edited by k. nagai. J Mol Biol (2000) 297:1183–94. doi: 10.1006/jmbi.2000.3642 10764582

[53] Goldfarb DS Corbett AH Mason DA Harreman MT Adam SA. Importin α: a multipurpose nuclear-transport receptor. Trends Cell Biol (2004) 14:505–14. doi: 10.1016/j.tcb.2004.07.016 15350979

[54] Terry LJ Shows EB Wente SR. Crossing the nuclear envelope: Hierarchical regulation of nucleocytoplasmic transport. Science (2007) 318:1412–6. doi: 10.1126/science.1142204 18048681

[55] Natalia F Celso C. Mechanisms and signals for the nuclear import of proteins. Curr Genomics (2009) 10:550–7. doi: 10.2174/138920209789503941 PMC2817886 20514217

[56] Marfori M Mynott A Ellis JJ Mehdi AM Saunders NF Curmi PM. Molecular basis for specificity of nuclear import and prediction of nuclear localization. Biochim Biophys Acta (2011) 1813:1562–77. doi: 10.1016/j.bbamcr.2010.10.013 20977914

[57] Miyamoto Y Yamada K Yoneda Y. Importin α: a key molecule in nuclear transport and non-transport functions. J Biochem (2016) 160:69–75. doi: 10.1093/jb/mvw036 27289017

[58] Panagiotopoulos AA Polioudaki C Ntallis SG Dellis D Notas G Panagiotidis CA. The sequence [EKRKI(E/R)(K/L/R/S/T)] is a nuclear localization signal for importin 7 binding (NLS7). Biochim Biophys Acta Gen Subj (2021) 1865:129851. doi: 10.1016/j.bbagen.2021.129851 33482249

[59] Garcia-Garcia M Sanchez-Perales S Jarabo P Calvo E Huyton T Fu L. Mechanical control of nuclear import by importin-7 is regulated by its dominant cargo YAP. Nat Commun (2022) 13:1174. doi: 10.1038/s41467-022-28693-y 35246520 PMC8897400

[60] Panagiotopoulos AA Kalyvianaki K Tsodoulou PK Darivianaki MN Dellis D Notas G. Recognition motifs for Importin 4 (LPPRS(G/P)P) and Importin 5 (KP(K/Y)LV) binding, by bio-informatic simulation and experimental *in vitro* validation. Comput Struct Biotechnol J (2022). 10.1016/j.csbj.2022.10.015 PMC9646746 36382187

[61] Goujon M Mcwilliam H Li W Valentin F Squizzato S Paern J. A new bioinformatics analysis tools framework at EMBL-EBI. Nucleic Acids Res (2010) 38:W695–699. doi: 10.1093/nar/gkq313 PMC2896090 20439314

[62] Sievers F Wilm A Dineen D Gibson TJ Karplus K Li W. Fast, scalable generation of high-quality protein multiple sequence alignments using clustal omega. Mol Syst Biol (2011) 7:539. doi: 10.1038/msb.2011.75 21988835 PMC3261699

[63] Baek M Dimaio F Anishchenko I Dauparas J Ovchinnikov S Lee GR. Accurate prediction of protein structures and interactions using a three-track neural network. Science (2021) 373:871–6. doi: 10.1126/science.abj8754 PMC7612213 34282049

[64] Jumper J Evans R Pritzel A Green T Figurnov M Ronneberger O. Highly accurate protein structure prediction with AlphaFold. Nature (2021) 596:583–9. doi: 10.1038/s41586-021-03819-2 PMC8371605 34265844

[65] Callaway E. What's next for AlphaFold and the AI protein-folding revolution. Nature (2022) 604:234–8. doi: 10.1038/d41586-022-00997-5 35418629

[66] Ko J Park H Heo L Seok C. GalaxyWEB server for protein structure prediction and refinement. Nucleic Acids Res (2012) 40:W294–297. doi: 10.1093/nar/gks493 PMC3394311 22649060

[67] Heo L Park H Seok C. GalaxyRefine: Protein structure refinement driven by side-chain repacking. Nucleic Acids Res (2013) 41:W384–388. doi: 10.1093/nar/gkt458 PMC3692086 23737448

[68] Shin W Lee G Heo L Lee H Seok C. Prediction of protein structure and interaction by GALAXY protein modeling programs. Bio Design (2014) 2:1–11.

[69] Lee GR Seok C. Galaxy7TM: flexible GPCR-ligand docking by structure refinement. Nucleic Acids Res (2016) 44:W502–506. doi: 10.1093/nar/gkw360 PMC4987912 27131365

[70] Colovos C Yeates TO. Verification of protein structures: patterns of nonbonded atomic interactions. Protein Sci (1993) 2:1511–9. doi: 10.1002/pro.5560020916 PMC2142462 8401235

[71] Barretina J Caponigro G Stransky N Venkatesan K Margolin AA Kim S. The cancer cell line encyclopedia enables predictive modelling of anticancer drug sensitivity. Nature (2012) 483:603–7. doi: 10.1038/nature11003 PMC3320027 22460905

[72] Nusinow DP Szpyt J Ghandi M Rose CM Mcdonald ER 3rd Kalocsay M. Quantitative proteomics of the cancer cell line encyclopedia. Cell (2020) 180:387–402 e316. doi: 10.1016/j.cell.2019.12.023 31978347 PMC7339254

[73] Kalyvianaki K Drosou I Notas G Castanas E Kampa M. Enhanced OXER1 expression is indispensable for human cancer cell migration. Biochem Biophys Res Commun (2021) 584:95–100. doi: 10.1016/j.bbrc.2021.11.024 34775286

[74] Wang R Brattain MG. The maximal size of protein to diffuse through the nuclear pore is larger than 60kDa. FEBS Lett (2007) 581:3164–70. doi: 10.1016/j.febslet.2007.05.082 PMC4064367 17588566

[75] Kocanova S Mazaheri M Caze-Subra S Bystricky K. Ligands specify estrogen receptor alpha nuclear localization and degradation. BMC Cell Biol (2010) 11:98. doi: 10.1186/1471-2121-11-98 21143970 PMC3009626

[76] Re A Colussi C Nanni S Aiello A Bacci L Grassi C. Nucleoporin 153 regulates estrogen-dependent nuclear translocation of endothelial nitric oxide synthase and estrogen receptor beta in prostate cancer. Oncotarget (2018) 9:27985–97. doi: 10.18632/oncotarget.25462 PMC6021351 29963256

[77] Freedman ND Yamamoto KR. Importin 7 and importin alpha/importin beta are nuclear import receptors for the glucocorticoid receptor. Mol Biol Cell (2004) 15:2276–86. doi: 10.1091/mbc.e03-11-0839 PMC404022 15004228

[78] Savory JG Hsu B Laquian IR Giffin W Reich T Hache RJ. Discrimination between NL1- and NL2-mediated nuclear localization of the glucocorticoid receptor. Mol Cell Biol (1999) 19:1025–37. doi: 10.1128/MCB.19.2.1025 PMC116033 9891038

[79] Tao T Lan J Lukacs GL Hache RJ Kaplan F. Importin 13 regulates nuclear import of the glucocorticoid receptor in airway epithelial cells. Am J Respir Cell Mol Biol (2006) 35:668–80. doi: 10.1165/rcmb.2006-0073OC 16809634

[80] Thornton JW. Evolution of vertebrate steroid receptors from an ancestral estrogen receptor by ligand exploitation and serial genome expansions. Proc Natl Acad Sci USA (2001) 98:5671–6. doi: 10.1073/pnas.091553298 PMC33271 11331759

[81] Gallo D Haddad I Duvillier H Jacquemotte F Laios I Laurent G. Trophic effect in MCF-7 cells of ERalpha17p, a peptide corresponding to a platform regulatory motif of the estrogen receptor alpha–underlying mechanisms. J Steroid Biochem Mol Biol (2008) 109:138–49. doi: 10.1016/j.jsbmb.2007.12.012 18262408

