# Supplementary material for: Importins involved in the nuclear transportation of steroid hormone receptors: In silico and in vitro data
Source: Front Endocrinol (Lausanne). 2022 Sep 6;13:954629. doi: 10.3389/fendo.2022.954629 (PMC9487861; doi:10.3389/fendo.2022.954629)
Supplement: Supplementary file 1 [file Presentation_1.pdf]

Importins involved in the nuclear transportation of steroid hormone receptors: *In silico* and *in vitro* data

Konstantina Kalyvianaki, Athanasios A. Panagiotopoulos, Maria Patentalaki, Elias Castanas, Marilena Kampa

Supplemental Material

Contents

Supplemental Tables 2
Supplemental Table 1 2
Supplemental Table 2 3
Supplemental Table 3 4
Supplemental Table 4 5
Supplemental Figures 6
Supplemental Figure 1 6
Supplemental Figure 2 13
Supplemental Figure 3 14
Supplemental Figure 4 15
Supplemental Figure 5 16
Supplemental Figure 6 18
Supplemental Figure 7 20
Supplemental Figure 8 22
Supplemental Figure 9 23
Supplemental Figure 10 24
References 25

Supplemental Tables

Supplemental Table 1

Accession (Uniprot) references and hinge region identification of the receptors used in this study

Receptor	Uniprot Reference	Hinge Region
Estrogen Receptor α (ESR1)	P03372.2	251-310
Estrogen Receptor β (ESR2)	Q92731.2	215-263
Androgen Receptor (AR)	P10275.3	633-688
Glucocorticoid Receptor (GR)	P04150.1	494-523
Progesterone Receptor (PR)	P06401.4	640-678
Mineralocorticoid Receptor (MR)	P08235.3	669-725

Supplemental Table 2

Crystals used for the identification of NLS sequences on steroid hormone receptors. PDB codes of the retained sequences are presented, as reported in the protein data bank (https://www.rcsb.org/).

**Receptor**	**A/B domain (AF1) (Predicted with AlphaFold)**	**C-domain (DBD) (Predicted with AlphaFold)**	**E/F domain (LBD, AF2)**		
			**Unliganded (Predicted with AlphaFold)**	**Liganded with Agonist**	**Liganded with Partial Agonist or Antagonist**
ERα	AF-P03372-F1	AF-P03372-F1	AF-P03372-F1	1A52 DOI: 10.2210/pdb1A52/pdb	1ERR DOI: 10.2210/pdb1ERR/pdb
ERβ	AF-Q92731-F1	AF-Q92731-F1	AF-Q92731-F1	5TOA DOI: 10.2210/pdb5TOA/pdb	1QKM DOI: 10.2210/pdb1QKM/pdb
PR	AF-P06401-F1	AF-P06401-F1	AF-P06401-F1	1A28 DOI: 10.2210/pdb1A28/pdb	2W8Y DOI: 10.2210/pdb2W8Y/pdb
AR	AF-P10275-F1	AF-P10275-F1	AF-P10275-F1	1T5Z DOI: 10.2210/pdb1T5Z/pdb	3B5R DOI: 10.2210/pdb3B5R/pdb
GR	AF-P04150-F1	AF-P04150-F1	AF-P04150-F1	4P6X DOI: 10.2210/pdb4P6X/pdb	1NHZ DOI: 10.2210/pdb1NHZ/pdb
MR	AF-P08235-F1	AF-P08235-F1	AF-P08235-F1	2AA2 DOI: 10.2210/pdb2AA2/pdb	5L7E DOI: 10.2210/pdb5L7E/pdb

Supplemental Table 3

Antibodies used for the different steroid receptors and Lamins of the nuclear envelope

Protein of interest	Primary antibody	Secondary antibody
**PR**	PR (C-19) rabbit polyclonal antibody: sc-538 (Santa Cruz Biotechnology, Inc.)<br>Dilution: 1:50	Donkey anti-rabbit IgG H&L Alexa Fluor® 647 (ab150075) (Abcam)<br>Dilution: 1:500
**ERα**	ERα (HC-184) rabbit polyclonal antibody: sc-7207 (Santa Cruz Biotechnology, Inc.)<br>Dilution: 1:50	Donkey anti-rabbit IgG H&L Alexa Fluor® 647 (ab150075) (Abcam)<br>Dilution: 1:500
**AR**	AR (411) mouse monoclonal antibody: sc-7305 (Santa Cruz Biotechnology, Inc.)<br>Dilution: 1:50	Goat anti-mouse IgG H&L Alexa CF® 555 (20030) (Biotium)<br>Dilution: 1:500
**Lamin B1**	Lamin B (M-20) goat polyclonal antibody: sc-6217-R (Santa Cruz Biotechnology, Inc.)<br>Dilution: 1:20	Mouse anti-goat IgG-FITC: sc-2356 (Santa Cruz Biotechnology, Inc.)<br>Dilution: 1:100
**Lamin A/C**	Lamin A/C (636) mouse monoclonal antibody: sc-7292 (Santa Cruz Biotechnology, Inc.)<br>Dilution: 1:20	Goat anti-mouse IgG H&L Alexa Fluor® 488 (A11001) (Invitrogen)<br>Dilution: 1:500

Supplemental Table 4

Importins and steroid receptors gene expression levels in T47D and LNCaP cells.

Gene expression levels of importins α, 4 and 5 (assayed by Real Time PCR), that their NLS sequences were recognized in the steroid receptors ERα, PR and AR. Data for the steroid receptor expression levels were obtained from *CCLE* database *(Cancer Cell Line Encyclopedia* https://sites.broadinstitute.org/ccle/) (Barretina et al., 2012).

	Gene Expression*		
CELL LINE	IPO4	IPO5	IPOA1
T47D	0.015	0.060	0.474
LNCaP	0.036	0.132	0.549
*Gene expression/Cyclophilin A expression			

	Gene Expression#		
CELL LINE	ERα	PR	AR
T47D	5.5	6.3	2.9
LNCaP	0.0	0.1	6.2
# Data from CCLE database			

Supplemental Figures

Supplemental Figure 1

ESTROGEN RECEPTOR ALPHA – P03372

```
        10         20         30         40         50
MTMTLHTKAS GMALLHQIQG NELEPLNRPQ LKIPLERPLG EVYLDSSKPA
        60         70         80         90        100
VYNYPEGAAY EFNAAAAANA QVYGQTGLPY GPGSEAAAFG SNGLGGFPPL
       110        120        130        140        150
NSVSPSPLML LHPPPQLSPF LQPHGQQVPY YLENEPSGYT VREAGPPAFY
       160        170        180        190        200
RPNSDNRRQG GRERLASTND KGSMAMESAK ETRYCAVCND YASGYHYGVW
                               K KKR-A
                               K RKR-A
       210        220        230        240        250
SCEGCKAFFK RSIQGHNDYM CPATNQCTID KNRRKSCQAC RLRKCYEVGM
                                 KKKR-A



       260        270        280        290        300
MKGGIRKDRR GGRMLKHKRQ RDDGEGRGEV GSAGDMRAAN LWPSPLMIKR
      KKKR-A    KKKR-A                              KR
     KKKR-A     KRKR-A                              KK
      KRKR-A                                        KR
                                                     K

                KRKK-A
                KKKK-A
       310        320        330        340        350
SKKNSLALSL TADQMVSALL DAEPPILYSE YDPTRPFSEA SMMGLLTNLA
KK-A
KK-A
KR-A
KKK-A

       360        370        380        390        400
DRELVHMINW AKRVPGFVDL TLHDQVHLLE CAWLEILMIG LVWRSMEHPG
       410        420        430        440        450
KLLFAPNLLL DRNQGKCVEG MVEIFDMLLA TSSRFRMMNL QGEEFVCLKS
       460        470        480        490        500
IILLNSGVYT FLSSTLKSLE EKDHIHRVLD KITDTLIHLM AKAGLTLQQQ
       510        520        530        540        550
HQRLAQLLLI LSHIRHMSNK GMEHLYSMKC KNVVPLYDLL LEMLDAHRLH
```

```
       560        570        580        590
APTSRGGASV EETDQSHLAT AGSTSSHSLQ KYYITGEAEG FPATV
```

ESTROGEN RECEPTOR BETA – Q92731

```
        10         20         30         40         50
MDIKNSPSSL NSPSSYNCSQ SILPLEHGSI YIPSSYVDSH HEYPAMTFYS
        60         70         80         90        100
PAVMNYSIPS NVTNLEGGPG RQTTSPNVLW PTPGHLSPLV VHRQLSHLYA
       110        120        130        140        150
EPQKSPWCEA RSLEHTLPVN RETLKRKVSG NRCASPVTGP GSKRDAHFCA

       160        170        180        190        200
VCSDYASGYH YGVWSCEGCK AFFKRSIQGH NDYICPATNQ CTIDKNRRKS

                                                KRKR-A
                                                KKKR-A

       210        220        230        240        250
CQACRLRKCY EVGMVKCGSR RERCGYRLVR RQRSADEQLH CAGKAKRSGG
                    K RKR-A    K RKR-A         KKKK-A
                                               KRKK-A
                                               KRKR-A
                                               KKKR-A
       260        270        280        290        300
HAPRVRELLL DALSPEQLVL TLLEAEPPHV LISRPSAPFT EASMMMSLTK
       310        320        330        340        350
LADKELVHMI SWAKKIPGFV ELSLFDQVRL LESCWMEVLM MGLMWRSIDH

       360        370        380        390        400
PGKLIFAPDL VLDRDEGKCV EGILEIFDML LATTSRFREL KLQHKEYLCV
       410        420        430        440        450
KAMILLNSSM YPLVTATQDA DSSRKLAHLL NAVTDALVWV IAKSGISSQQ

       460        470        480        490        500
QSMRLANLLM LLSHVRHASN KGMEHLLNMK CKNVVPVYDL LLEMLNAHVL
       510        520        530
RGCKSSITGS ECSPAEDSKS KEGSQNPQSQ
                   KK KK-A
                   KR KK-A
```

ANDROGEN RECEPTOR-P10275

```
        10         20         30         40         50
MEVQLGLGRV YPRPPSKTYR GAFQNLFQSV REVIQNPGPR HPEAASAAPP
                 KRKR-A
        60         70         80         90        100
GASLLLLQQQ QQQQQQQQQQ QQQQQQQQQQ ETSPRQQQQQ QGEDGSPQAH
       110        120        130        140        150
RRGPTGYLVL DEEQQPSQPQ SALECHPERG CVPEPGAAVA ASKGLPQQLP
       160        170        180        190        200
APPDEDDSAA PSTLSLLGPT FPGLSSCSAD LKDILSEAST MQLLQQQQQE
       210        220        230        240        250
AVSEGSSSGR AREASGAPTS SKDNYLGGTS TISDNAKELC KAVSVSMGLG
       260        270        280        290        300
VEALEHLSPG EQLRGDCMYA PLLGVPPAVR PTPCAPLAEC KGSLLDDSAG
       310        320        330        340        350
KSTEDTAEYS PFKGGYTKGL EGESLGCSGS AAAGSSGTLE LPSTLSLYKS
       360        370        380        390        400
GALDEAAAYQ SRDYYNFPLA LAGPPPPPPP PHPHARIKLE NPLDYGSAWA
                        LPPRSPP-4
       410        420        430        440        450
AAAAQCRYGD LASLHGAGAA GPGSGSPSAA ASSSWHTLFT AEEGQLYGPC
       460        470        480        490        500
GGGGGGGGGG GGGGGGGGGG GGGEAGAVAP YGYTRPPQGL AGQESDFTAP
       510        520        530        540        550
DVWYPGGMVS RVPYPSPTCV KSEMGPWMDS YSGPYGDMRL ETARDHVLPI
       560        570        580        590        600
DYYFPPQKTC LICGDEASGC HYGALTCGSC KVFFKRAAEG KQKYLCASRN

       610        620        630        640        650
DCTIDKFRRK NCPSCRLRKC YEAGMTLGAR KLKKLGNLKL QEEGEASSTT
                                  KKKK-A
     KRKR-A                     K KKK-A

       660        670        680        690        700
SPTEETTQKL TVSHIEGYEC QPIFLNVLEA IEPGVVCAGH DNNQPDSFAA
       710        720        730        740        750
LLSSLNELGE RQLVHVVKWA KALPGFRNLH VDDQMAVIQY SWMGLMVFAM
       760        770        780        790        800
GWRSFTNVNS RMLYFAPDLV FNEYRMHKSR MYSQCVRMRH LSQEFGWLQI

       810        820        830        840        850
TPQEFLCMKA LLLFSIIPVD GLKNQKFFDE LRMNYIKELD RIIACKRKNP
                        KKKK-A
       860        870        880        890        900
TSCSRRFYQL TKLLDSVQPI ARELHQFTFD LLIKSHMVSV DFPEMMAEII
```

```
        910        920
SVQVPKILSG KVKPIYFHTQ
```

Glucocorticoid receptor-P04150

```
        10         20         30         40         50
MDSKESLTPG REENPSSVLA QERGDVMDFY KTLRGGATVK VSASSPSLAV
                                 KRKR-A
                                 KKKR-A
        60         70         80         90        100
ASQSDSKQRR LLVDFPKGSV SNAQQPDLSK AVSLSMGLYM GETETKVMGN
      KRKR-A               KPKLV-5
                           KPYLV-5
       110        120        130        140        150
DLGFPQQGQI SLSSGETDLK LLEESIANLN RSTSVPENPK SSASTAVSAA

       160        170        180        190        200
PTEKEFPKTH SDVSSEQQHL KGQTGTNGGN VKLYTTDQST FDILQDLEFS

       210        220        230        240        250
SGSPGKETNE SPWRSDLLID ENCLLSPLAG EDDSFLLEGN SNEDCKPLIL
                                                 KPKLV-5
                                                 KPYLV-5
       260        270        280        290        300
PDTKPKIKDN GDLVLSSPSN VTLPQVKTEK EDFIELCTPG VIKQEKLGTV
                            KKKK-A                KKKK-A

   KPKLV-5
       310        320        330        340        350
YCQASFPGAN IIGNKMSAIS VHGVSTSGGQ MYHYDMNTAS LSQQQDQKPI

       360        370        380        390        400
FNVIPPIPVG SENWNRCQGS GDDNLTSLGT LNFPGRTVFS NGYSSPSMRP
       410        420        430        440        450
DVSSPPSSSS TATTGPPPKL CLVCSDEASG CHYGVLTCGS CKVFFKRAVE
       460        470        480        490        500
GQHNYLCAGR NDCIIDKIRR KNCPACRYRK CLQAGMNLEA RKTKKKIKGI
                                                KKKK-A
                                                KKKK-A
                 KRKR-A
                                               KRKK-A
                                                 KPKLV-5
                                           LPPRSPP-7
       510        520        530        540        550
QQATTGVSQE TSENPGNKTI VPATLPQLTP TLVSLLEVIE PEVLYAGYDS
                              KP KLV-5
                              KP YLV-5

       560        570        580        590        600
SVPDSTWRIM TTLNMLGGRQ VIAAVKWAKA IPGFRNLHLD DQMTLLQYSW
       610        620        630        640        650
MFLMAFALGW RSYRQSSANL LCFAPDLIIN EQRMTLPCMY DQCKHMLYVS


       660        670        680        690        700
SELHRLQVSY EEYLCMKTLL LLSSVPKDGL KSQELFDEIR MTYIKELGKA
```

```
       710        720        730        740        750
IVKREGNSSQ NWQRFYQLTK LLDSMHEVVE NLLNYCFQTF LDKTMSIEFP

       760        770
EMLAEIITNQ IPKYSNGNIK KLLFHQK
```

PROGESTERONE RECEPTOR – P06401

```
        10         20         30         40         50
MTELKAKGPR APHVAGGPPS PEVGSPLLCR PAAGPFPGSQ TSDTLPEVSA
        60         70         80         90        100
IPISLDGLLF PRPCQGQDPS DEKTQDQQSL SDVEGAYSRA EATRGAGGSS
       110        120        130        140        150
SSPPEKDSGL LDSVLDTLLA PSGPGQSQPS PPACEVTSSW CLFGPELPED
       160        170        180        190        200
PPAAPATQRV LSPLMSRSGC KVGDSSGTAA AHKVLPRGLS PARQLLLPAS
              LPPRSG-4
       210        220        230        240        250
ESPHWSGAPV KPSPQAAAVE VEEEDGSESE ESAGPLLKGK PRALGGAAAG
       260        270        280        290        300
GGAAAVPPGA AAGGVALVPK EDSRFSAPRV ALVEQDAPMA PGRSPLATTV
       310        320        330        340        350
MDFIHVPILP LNHALLAART RQLLEDESYD GGAGAASAFA PPRSSPCASS
       360        370        380        390        400
TPVAVGDFPD CAYPPDAEPK DDAYPLYSDF QPPALKIKEE EEGAEASARS
                                       KKKK-A
       410        420        430        440        450
PRSYLVAGAN PAAFPDFPLG PPPPLPPRAT PSRPGEAAVT AAPASASVSS
                          LPPRSG P-4
KPYLV-5
       460        470        480        490        500
ASSSGSTLEC ILYKAEGAPP QQGPFAPPPC KAPGASGCLL PRDGLPSTSA
                                         LP PRSGP-4

       510        520        530        540        550
SAAAAGAAPA LYPALGLNGL PQLGYQAAVL KEGLPQVYPP YLNYLRPDSE
                                    LPPRSPP-4
       560        570        580        590        600
ASQSPQYSFE SLPQKICLIC GDEASGCHYG VLTCGSCKVF FKRAMEGQHN

       610        620        630        640        650
YLCAGRNDCI VDKIRRKNCP ACRLRKCCQA GMVLGGRKFK KFNKVRVVRA
                                        KKK K-A
       660        670        680        690        700
LDAVALPQPV GVPNESQALS QRFTFSPGQD IQLIPPLINL LMSIEPDVIY

       710        720        730        740        750
AGHDNTKPDT SSSLLTSLNQ LGERQLLSVV KWSKSLPGFR NLHIDDQITL
```

```
                                        KRKK-A
         760        770        780        790        800
IQYSWMSLMV FGLGWRSYKH VSGQMLYFAP DLILNEQRMK ESSFYSLCLT
                                K RKK-A

         810        820        830        840        850
MWQIPQEFVK LQVSQEEFLC MKVLLLLNTI PLEGLRSQTQ FEEMRSSYIR
         860        870        880        890        900
ELIKAIGLRQ KGVVSSSQRF YQLTKLLDNL HDLVKQLHLY CLNTFIQSRA
         910        920        930
LSVEFPEMMS EVIAAQLPKI LAGMVKPLLF HKK
```

MINERALOCORTICOID RECEPTOR – P08235

```
          10         20         30         40         50
METKGYHSLP EGLDMERRWG QVSQAVERSS LGPTERTDEN NYMEIVNVSC
          60         70         80         90        100
VSGAIPNNST QGSSKEKQEL LPCLQQDNNR PGILTSDIKT ELESKELSAT
                KKKK-A
         110        120        130        140        150
VAESMGLYMD SVRDADYSYE QQNQQGSMSP AKIYQNVEQL VKFYKGNGHR
         160        170        180        190        200
PSTLSCVNTP LRSFMSDSGS SVNGGVMRAV VKSPIMCHEK SPSVCSPLNM
         210        220        230        240        250
TSSVCSPAGI NSVSSTTASF GSFPVHSPIT QGTPLTCSPN VENRGSRSHS
                                             EKRKIRS-7
         260        270        280        290        300
PAHASNVGSP LSSPLSSMKS SISSPPSHCS VKSPVSSPNN VTLRSSVSSP
         310        320        330        340        350
ANINNSRCSV SSPSNTNNRS TLSSPAASTV GSICSPVNNA FSYTASGTSA
         360        370        380        390        400
GSSTLRDVVP SPDTQEKGAQ EVPFPKTEEV ESAISNGVTG QLNIVQYIKP
         410        420        430        440        450
EPDGAFSSSC LGGNSKINSD SSFSVPIKQE STKHSCSGTS FKGNPTVNPF
         460        470        480        490        500
PFMDGSYFSF MDDKDYYSLS GILGPPVPGF DGNCEGSGFP VGIKQEPDDG
         510        520        530        540        550
SYYPEASIPS SAIVGVNSGG QSFHYRIGAQ GTISLSRSAR DQSFQHLSS
         560        570        580        590        600
PPVNTLVESW KSHGDLSSRR SDGYPVLEYI PENVSSSTLR SVSTGSSRPS
         610        620        630        640        650
KICLVCGDEA SGCHYGVVTC GSCKVFFKRA VEGQHNYLCA GRNDCIIDKI


         660        670        680        690        700
RRKNCPACRL QKCLQAGMNL GARKSKKLGK LKGIHEEQPQ QQQPPPPPPP
                         KKKK-A
         710        720        730        740        750
PQSPEEGTTY IAPAKEPSVN TALVPQLSTI SRALTPSPVM VLENIEPEIV
```

```
        760        770        780        790        800
YAGYDSSKPD TAENLLSTLN RLAGKQMIQV VKWAKVLPGF KNLPLEDQIT
        810        820        830        840        850
LIQYSWMCLS SFALSWRSYK HTNSQFLYFA PDLVFNEEKM HQSAMYELCQ
        860        870        880        890        900
GMHQISLQFV RLQLTFEEYT IMKVLLLLST IPKDGLKSQA AFEEMRTNYI
        910        920        930        940        950
KELRKMVTKC PNNSGQSWQR FYQLTKLLDS MHDLVSDLLE FCFYTFRESH
        960        970        980
ALKVEFPAML VEIISDQLPK VESGNAKPLY FHRK
```

Identification of NLS sequences for Importins alpha (KRRR and KRKXK, olive green color), 4 ((L)PPRS(G/P)P, blue color), 5 (KP(K/Y)LV, pink color) and 7 (EKRKI(E/R)(K/L/R/S/T), red color) in the structures of estrogen receptors α and β (ERα and ERβ), progesterone receptor (PR), androgen receptor (AR), glucocorticoid receptor (GR) and mineralocorticoid receptor (MR). Multiple alignments were performed with the Cruscal Omega tool on the EMBL-EBI server (https://www.ebi.ac.uk/Tools/msa/clustalo/) (Goujon et al., 2010;Sievers et al., 2011). Only sequences with at least 50% sequence homology are shown. After the identified sequence the type of importin NLS is denoted (A, 4, 5, 7). The hinge region of each receptor is highlighted in grey color, while the protein reference of each receptor follows its name in the header.

Supplemental Figure 2

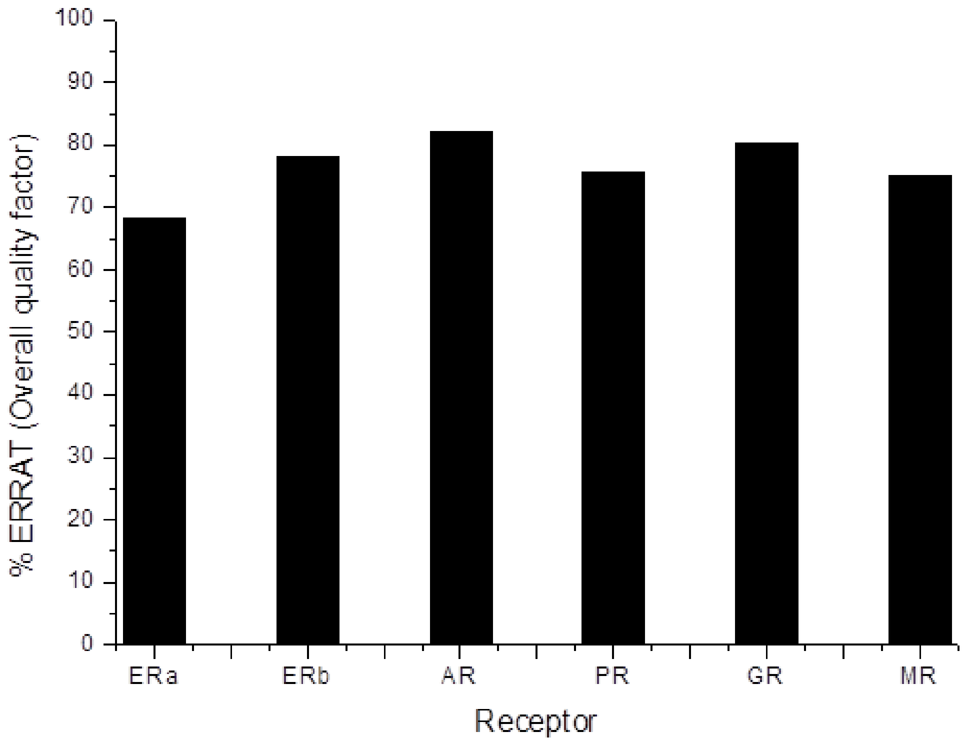

Supplemental Figure 2:

Total error of the structure comparison of the receptors used in this work, using the ERRAT2 web server (https://saves.mbi.ucla.edu/) (Colovos and Yeates, 1993) and a 9-residue sliding window. As shown, in all cases, the predicted error is lower that 95%, suggesting a correct conformation of the receptors.

Supplemental Figure 3

ERα Alpha Fold for N-Terminal

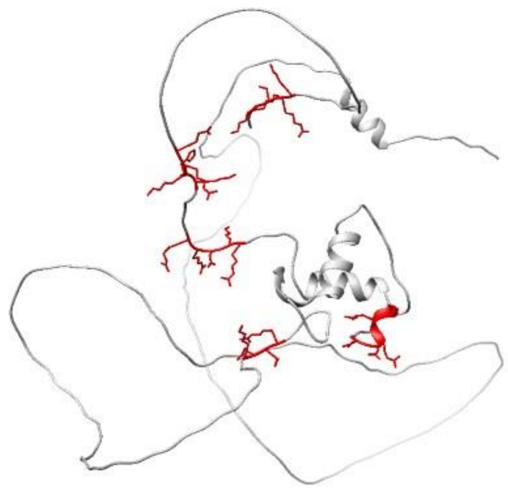

NLS Site	Active or Inactive
180-183	Active
231-235	Active
256-260	Active
265-271	Active
299-303	Active

Supplemental Figure 3

In silico prediction of the active or inactive state of the different NLS sequences on ERα. NLS sequences are shown in red sticks on the receptor structure. When they are present at the surface of the receptor 3D structure, they are characterized as "active" (i.e accessible to importins for binding). In contrast, when they are buried in the structure, away from the surface, they are inaccessible to importins and characterized as "inactive". Note that no NLS sequence was identified in the LBD of the receptor. Please, refer to Supplemental Figure 1 for the specificity of the identified sequences and in the Material and Methods section of the main text, for additional details.

Supplemental Figure 4

ERβ Alpha Fold for N-Terminal

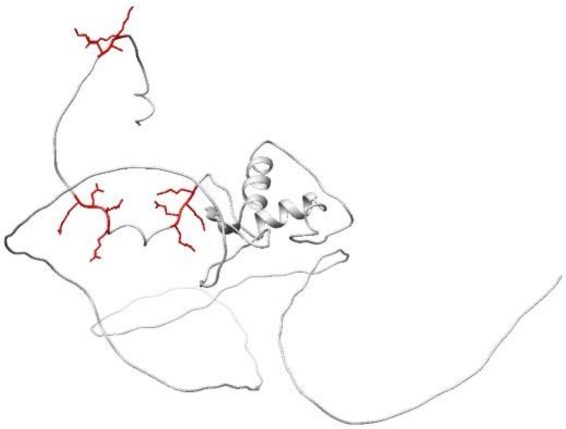

AA 1-259

NLS Site	Active or Inactive
220-223	Active
230-233	Active
244-247	Active

Supplemental Figure 4

In silico prediction of the active or inactive state of the different NLS sequences on ERβ. NLS sequences are shown in red sticks on the receptor structure. When they are present at the surface of the receptor 3D structure, they are characterized as "active" (i.e accessible to importins for binding). In contrast, when they are buried in the structure, away from the surface, they are inaccessible to importins and characterized as "inactive". Note that no NLS sequence was identified in the LBD of the receptor. Please, refer to Supplemental Figure 1 for the specificity of the identified sequences and in the Material and Methods section of the main text, for additional details.

Supplemental Figure 5

AR

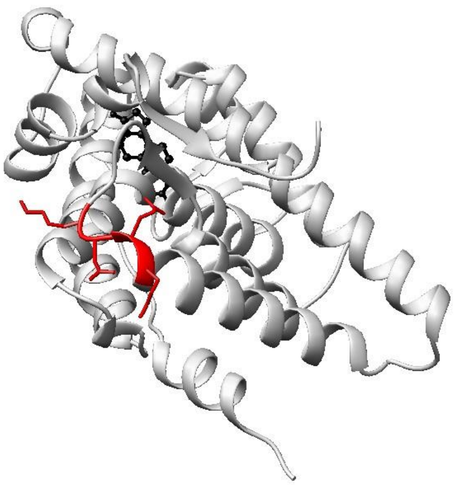

AA 669-918
Agonist

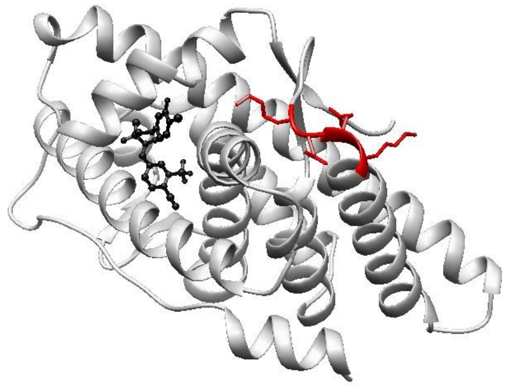

AA 671-917
Antagonist

NLS Site	Agonist	Antagonist
823-826	Active	Active

AR C-Terminal unliganded

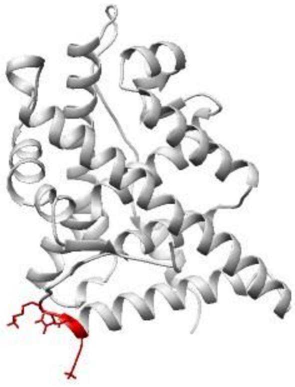

AA 669-918

NLS Site	Active or Inactive
823-826	Active

AR Alpha Fold for N -Terminal

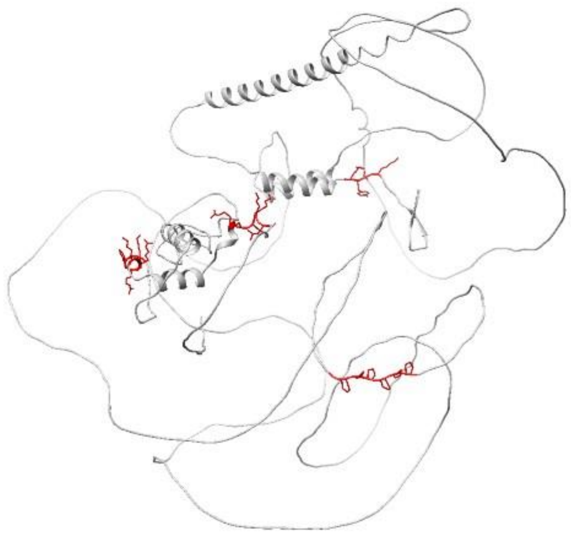

NLS Site	Active or Inactive
17-20	Active
373-379	Active
606-610	Active
630-635	Active

Supplemental Figure 5

In silico prediction of the active or inactive state of the different NLS sequences on the AR. NLS sequences are shown in red sticks on the receptor structure. When they are present at the surface of the receptor 3D structure, they are characterized as "active" (i.e accessible to importins for binding). In contrast, when they are buried in the structure, away from the surface, they are inaccessible to importins and characterized as "inactive". Please, refer to Supplemental Figure 1 for the specificity of the identified sequences and in the Material and Methods section of the main text, for additional details.

Supplemental Figure 6

Progesterone Receptor

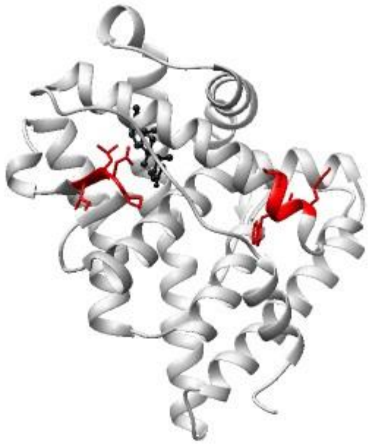

AA 682-932
Agonist

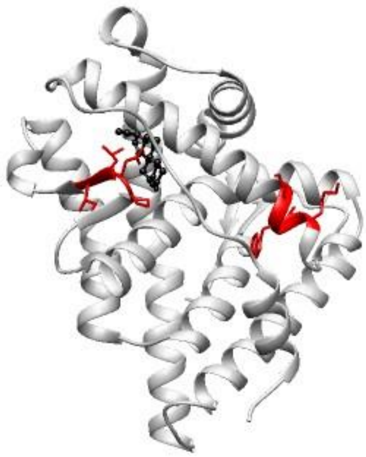

AA 683-933
Antagonist

NLS Site	Agonist	Antagonist
731-734	Inactive	Active
780-783	Active	Active

Progesterone Receptor C -Terminal unliganded

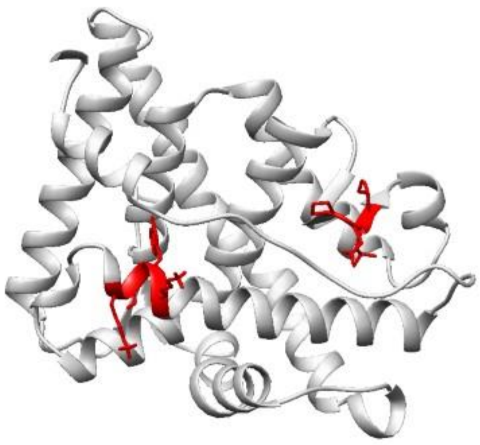

AA 682-932

NLS Site	Active or Inactive
731-734	Active
780-783	Active

Progesterone Receptor Alpha Fold for N- Terminal

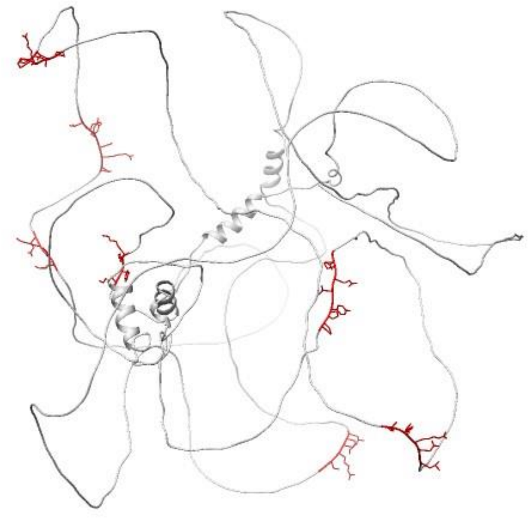

AA 1-681

NLS Site	Active or Inactive
165-169	Active
386-389	Active
401-406	Active
425-431	Active
489-495	Active
534-540	Active
638-641	Active

Supplemental Figure 6

In silico prediction of the active or inactive state of the different NLS sequences on the PR. NLS sequences are shown in red sticks on the receptor structure. When they are present at the surface of the receptor 3D structure, they are characterized as “active” (i.e accessible to importins for binding). In contrast, when they are buried in the structure, away from the surface, they are inaccessible to importins and characterized as “inactive”. Please, refer to Supplemental Figure 1 for the specificity of the identified sequences and in the Material and Methods section of the main text, for additional details.

Supplemental Figure 7

Glucocorticoid Receptor

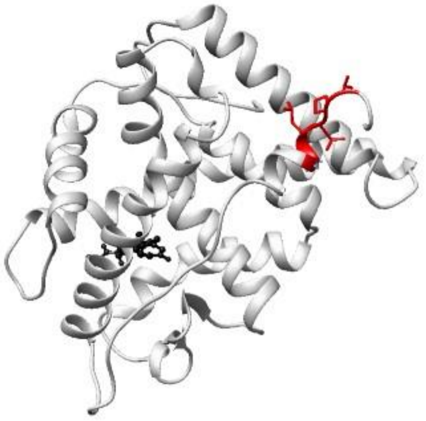

AA 526-777
Agonist

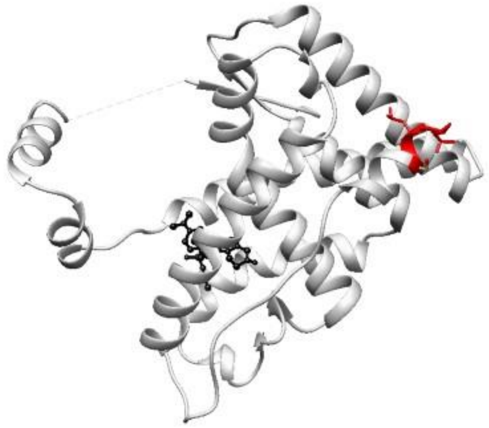

AA 530-776
Antagonist

NLS Site	Agonist	Antagonist
529-533	Active	Active

Glucocorticoid Receptor C-Terminal unliganded

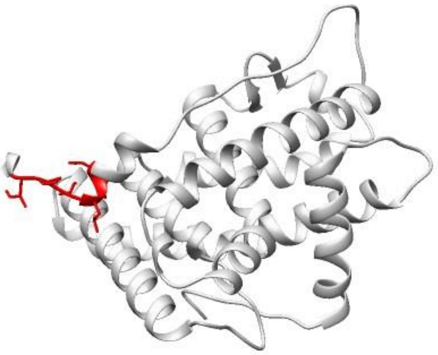

AA 526-777

NLS Site	Active or Inactive
529-534	Active

Glucocorticoid Receptor Alpha Fold for N-Terminal

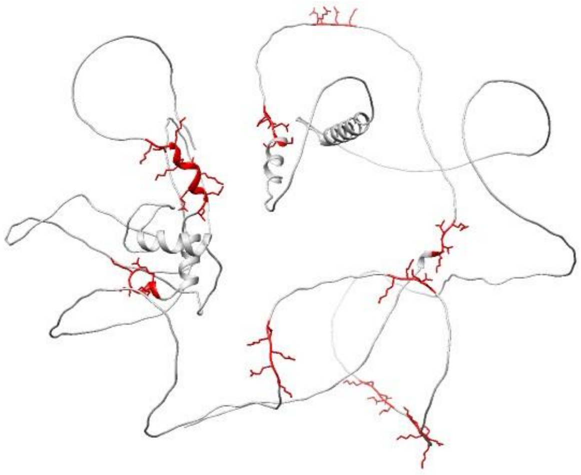

AA 1-525

NLS Site	Active or Inactive
31-34	Active
57-60	Active
75-79	Active
246-250	Active
253-258	Active
277-280	Active
292-296	Active
477-480	Active
488-498	Active

Supplemental Figure 7

In silico prediction of the active or inactive state of the different NLS sequences on the GR. NLS sequences are shown in red sticks on the receptor structure. When they are present at the surface of the receptor 3D structure, they are characterized as "active" (i.e accessible to importins for binding). In contrast, when they are buried in the structure, away from the surface, they are inaccessible to importins and characterized as "inactive". Please, refer to Supplemental Figure 1 for the specificity of the identified sequences and in the Material and Methods section of the main text, for additional details.

Supplemental Figure 8

Mineralocorticoid Receptor Alpha Fold for N- Terminal

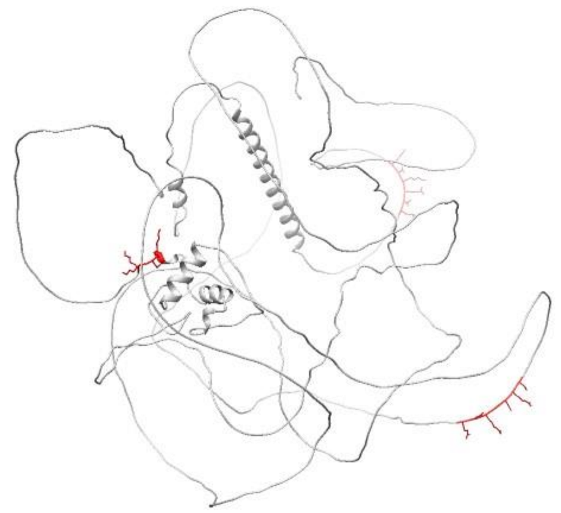

NLS Site	Active or Inactive
65-70	Active
242-248	Active
673-677	Active

Supplemental Figure 8

In silico prediction of the active or inactive state of the different NLS sequences on the MR. NLS sequences are shown in red sticks on the receptor structure. When they are present at the surface of the receptor 3D structure, they are characterized as “active” (i.e accessible to importins for binding). In contrast, when they are buried in the structure, away from the surface, they are inaccessible to importins and characterized as “inactive”. Note that no NLS sequence was identified in the LBD of the receptor. Please, refer to Supplemental Figure 1 for the specificity of the identified sequences and in the Material and Methods section of the main text, for additional details.

Supplemental Figure 9

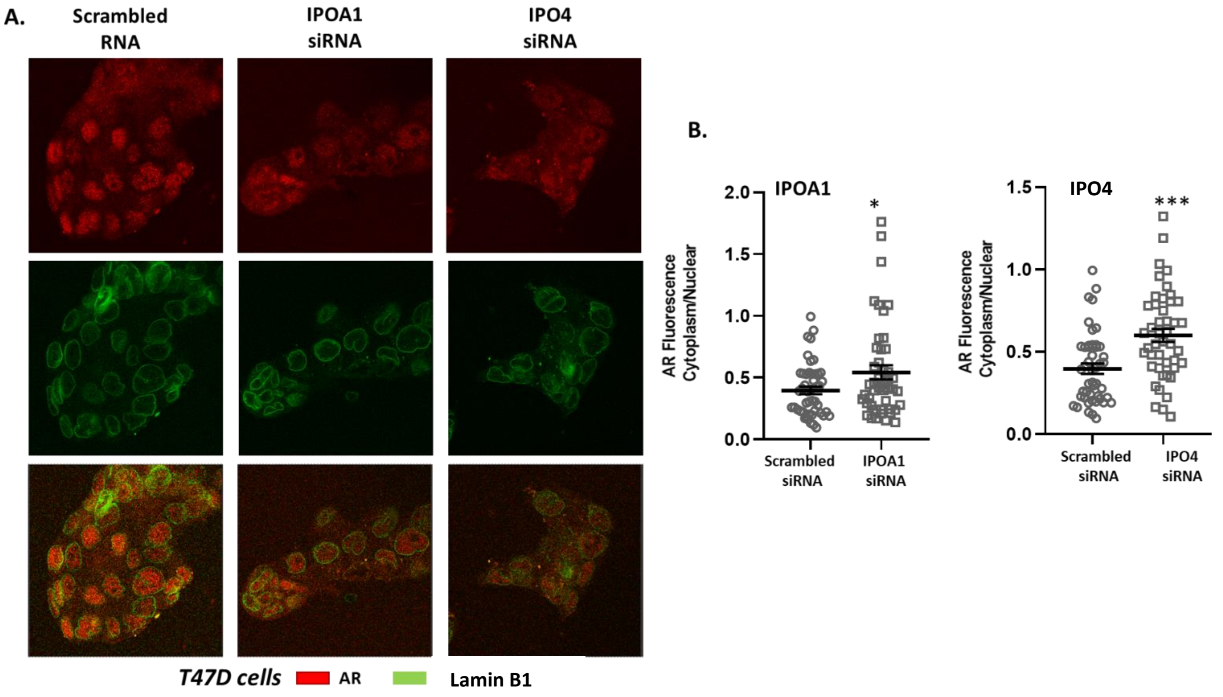

Supplemental Figure 9

A. Representative confocal pictures of T47D cells stained for AR (red) and lamin B1 (green) (3rd raw is their overlay). T47D cells were transfected either with a scrambled siRNA or a specific siRNA for importin α (IPOA1) or importin 4 (IPO4) and treated with DHT (10^{-7}M) for 90 min to ensure nuclear localization. Magnification x1260. **B.** Intensity of fluorescence in the cytoplasm and nucleus was quantified (see Material and Methods for details) in at least 50 cells per condition and is given as the Cytoplasm/Nuclear fluorescence ratio comparing cells with specific IPOA1 or IPO4 siRNA to those with the scrambled siRNA. * denotes statistical significance P< 0.05 and *** P< 0.001.

Supplemental Figure 10

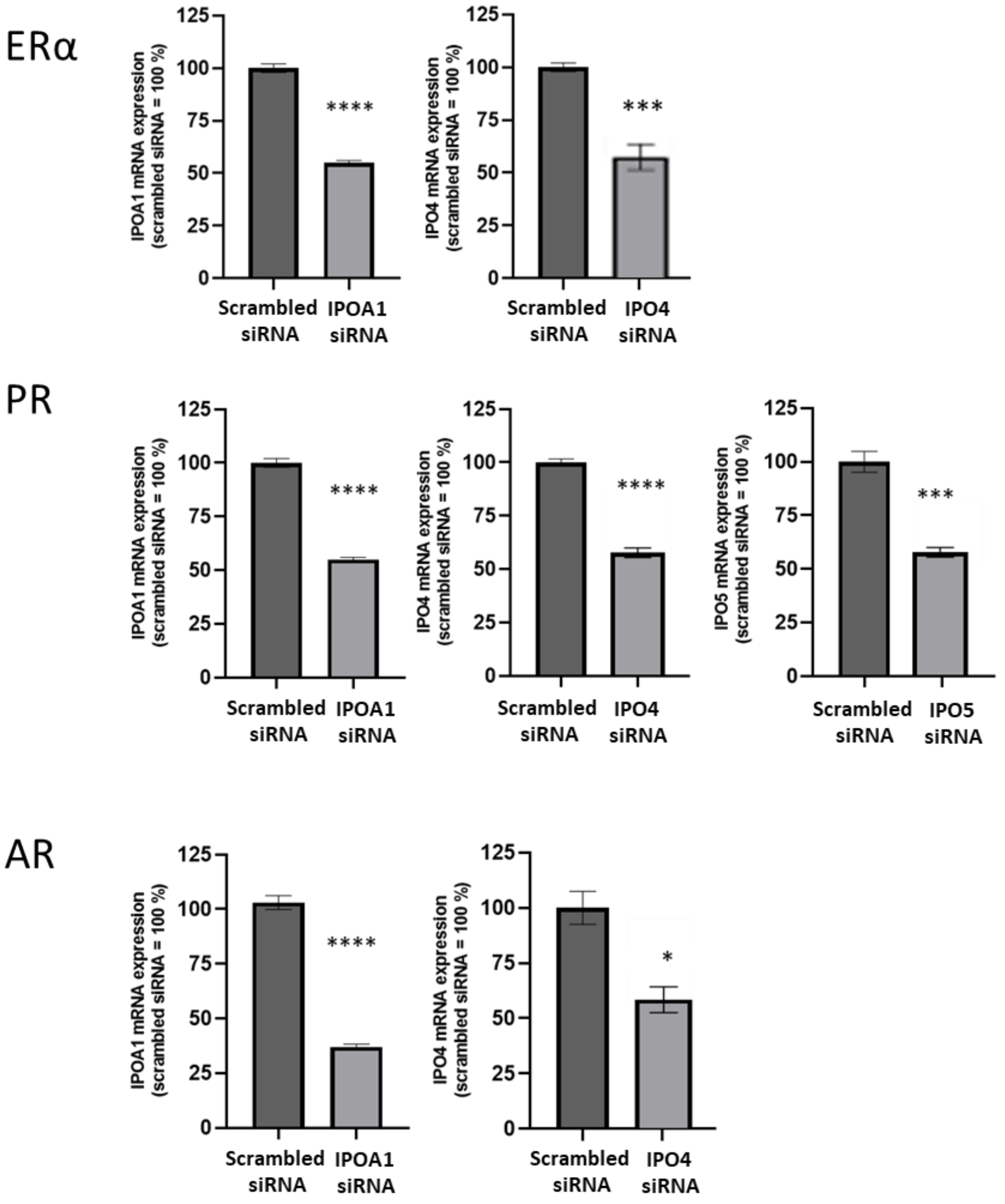

Supplemental Figure 10

Steroid receptor levels, (ERα and PR in T47D cells and AR in LNCaP cells) in cells transfected either with a scrambled siRNA or a specific siRNA for importin α (IPOA1), importin 4 (IPO4) or importin 5 (IPO5) ***** denotes statistical significance P< 0.05, *** P< 0.001 and ******** P< 0.0001.

References

Barretina, J., Caponigro, G., Stransky, N., Venkatesan, K., Margolin, A.A., Kim, S., Wilson, C.J., Lehar, J., Kryukov, G.V., Sonkin, D., Reddy, A., Liu, M., Murray, L., Berger, M.F., Monahan, J.E., Morais, P., Meltzer, J., Korejwa, A., Jane-Valbuena, J., Mapa, F.A., Thibault, J., Bric-Furlong, E., Raman, P., Shipway, A., Engels, I.H., Cheng, J., Yu, G.K., Yu, J., Aspesi, P., Jr., De Silva, M., Jagtap, K., Jones, M.D., Wang, L., Hatton, C., Palescandolo, E., Gupta, S., Mahan, S., Sougnez, C., Onofrio, R.C., Liefeld, T., Macconaill, L., Winckler, W., Reich, M., Li, N., Mesirov, J.P., Gabriel, S.B., Getz, G., Ardlie, K., Chan, V., Myer, V.E., Weber, B.L., Porter, J., Warmuth, M., Finan, P., Harris, J.L., Meyerson, M., Golub, T.R., Morrissey, M.P., Sellers, W.R., Schlegel, R., and Garraway, L.A. (2012). The Cancer Cell Line Encyclopedia enables predictive modelling of anticancer drug sensitivity. *Nature* 483**,** 603-607.

Colovos, C., and Yeates, T.O. (1993). Verification of protein structures: patterns of nonbonded atomic interactions. *Protein Sci* 2**,** 1511-1519.

Goujon, M., Mcwilliam, H., Li, W., Valentin, F., Squizzato, S., Paern, J., and Lopez, R. (2010). A new bioinformatics analysis tools framework at EMBL-EBI. *Nucleic Acids Res* 38**,** W695-699.

Sievers, F., Wilm, A., Dineen, D., Gibson, T.J., Karplus, K., Li, W., Lopez, R., Mcwilliam, H., Remmert, M., Soding, J., Thompson, J.D., and Higgins, D.G. (2011). Fast, scalable generation of high-quality protein multiple sequence alignments using Clustal Omega. *Mol Syst Biol* 7**,** 539.
